# Hyaluronic acid: A novel approach in regenerative/reconstructive periodontal therapy?

**DOI:** 10.1111/prd.12644

**Published:** 2025-08-18

**Authors:** Andrea Pilloni, Yoshinori Shirakata, Lorenzo Marini, Darko Božić, Richard J. Miron, Roberto Rotundo, Andreas Stavropoulos, Anton Sculean

**Affiliations:** ^1^ Section of Periodontology, Department of Oral and Maxillofacial Sciences Sapienza University of Rome Rome Italy; ^2^ Department of Periodontology Kagoshima University Graduate School of Medical and Dental Sciences Kagoshima Japan; ^3^ Department of Periodontology School of Dental Medicine, University of Zagreb Zagreb Croatia; ^4^ Department of Periodontology University of Bern Bern Switzerland; ^5^ Section of Periodontology School of Dentistry, University Vita e Salute Milan Italy; ^6^ Periodontology, Faculty of Odontology University of Malmö Malmö Sweden

**Keywords:** hyaluronan, hyaluronic acid, periodontics, periodontitis, periodontium, regeneration, wound healing

## Abstract

**Background:**

Although hyaluronic acid (HA) has long been used for many medical applications, only in recent years has it gained greater popularity in the field of periodontics because of its biological effects during wound healing. Even today, most clinicians are not aware that more than one type of HA exists and that the extent of its biological functions may vary depending upon the particular characteristics of the biomolecule itself.

**Aim:**

To review and synthesize the current preclinical and clinical evidence on the biological effects and therapeutic applications of HA in periodontology, with a focus on its role in wound healing and regeneration.

**Materials and methods:**

The origin and chemical structure of HA are discussed first, with a focus on the importance of its molecular weight and the possibility of modifying its structure and form. The main biological properties of HA followed by its effects on the cells of periodontal tissues are summarized and followed by the presentation of the results from preclinical studies in animals which have evaluated the effects of HA in various types of defects. Subsequently, the data from clinical studies evaluating the application of HA in nonsurgical periodontal therapy, regenerative periodontal surgery, and mucogingival surgery are summarized, and recommendations for the clinicians are provided.

**Results:**

The preclinical and clinical evidence indicates that HA accelerates the wound healing process through inflammatory mechanisms and enhances blood clot stability when applied to the root surface. It also influences the expression of both mineralized tissue markers and cementoblast‐specific genes, suggesting a potential role in cementum regeneration. HA strongly promotes osteoprogenitor growth while maintaining stemness, potentially regulating the balance between self‐renewal and differentiation during bone regeneration. Additionally, HA enhances periodontal ligament (PDL) cell adhesion and proliferation. It has been shown to improve the proliferative and migratory abilities of cells while inducing the expression of collagen type III alpha 1 (COL3A1) and TGFβ‐3 genes, which are characteristic of scarless fetal wound healing. Certain HA formulations upregulate the expression of genes encoding platelet‐derived growth factor B (PDGFB), fibroblast growth factor 2 (FGF‐2), and epidermal growth factor (EGF), all of which play crucial roles in the healing process. Histologic evidence from animal studies suggests that HA may promote periodontal regeneration when applied both non‐surgically and surgically—particularly in intrabony defects, gingival recessions, and, to some extent, in furcation defects. The data from clinical studies revealed that HA leads to statistically significant and clinically relevant improvements of probing depths and clinical attachment levels when used in conjunction with nonsurgical periodontal therapy and surgical therapy in intrabony and recession defects.

**Conclusion:**

The available data from preclinical and clinical studies provide robust evidence on the effects of HA to enhance periodontal wound healing and regeneration, and on the improved clinical outcomes when HA is used in conjunction with nonsurgical periodontal therapy and regenerative surgery in intrabony and recession defects.

## 1 INTRODUCTION

Hyaluronic acid (HA), though long used in medicine, has recently gained attention in periodontics for its beneficial role in wound healing. Its clinical effects vary depending on molecular characteristics, yet many practitioners remain unaware of the different HA types and their distinct properties. Periodontal therapy, as outlined by the European Federation of Periodontology, involves a stepwise approach: initial behavior changes and supragingival biofilm control, followed by subgingival debridement, and—if needed—re‐instrumentation or surgery.^1^ HA has been explored as an adjunct in both nonsurgical and surgical phases, as well as in mucogingival procedures, due to its regenerative potential.

This review summarizes HA's origin, structure, and biological properties, along with current evidence from in vitro, animal, and clinical studies in various periodontal applications.

## 2 HYALURONIC ACID: ORIGIN AND HISTORICAL BACKGROUND

The first report referencing HA dates back to 1880, when the French chemist Portes observed that the mucin in the vitreous humor, which he named hyalomucoid, behaved differently than other mucoids, such as those in the cornea and cartilage.^2^ Subsequently, Carl Morner of Sweden confirmed Portes' results in 1884, demonstrating that hyalomucoid possessed considerably less sulfur than mucoids isolated from the other two aforementioned tissues.^3^

Hyaluronic acid was first discovered in 1934 by Karl Meyer and his colleague and assistant John Palmer, both of whom were scientists at Columbia University, New York. They described a procedure for isolating a novel chemical substance from the vitreous humor of bovine eyes. They soon proposed the name hyaluronic acid, derived from the Greek word *hyalos* (glass), as the structure contained two sugar molecules, one of which was uronic acid, as follows: “we propose for convenience, the name ‘hyaluronic acid,’ from hyaloid (glassy, vitreous) + uronic acid.”^4^ The chemical structure of HA was determined by Weissman and Meyer 20 years later, in 1954.^5^ In 1986, the alternative name “hyaluronan” was proposed because at physiological pH, the carboxyl groups of the molecule are deprotonated and attract cations (e.g., Na^+^).^6^ HA was first applied for medicinal purposes in humans during eye surgery vitreous replacement, wherein the HA used was isolated from a human umbilical cord.^7^ Currently, HA can be industrially manufactured from animal sources and subsequently prepared for a variety of medical, pharmaceutical, nutritional and cosmetic applications.

## 3 MOLECULAR STRUCTURE AND PROPERTIES

HA is a linear glycosaminoglycan (GAG), also termed mucopolysaccharide, whose chemical structure is (C_14_H_21_NO_1_)_n_. It is composed of repeating units of d‐glucuronic acid and *N*–acetyl‐d‐glucosamine. The two saccharides are linked alternately by β‐1,4‐ and β‐1,3‐glycosidic bonds. This molecule has peculiar properties, including being negatively charged (due to the carboxylate groups), water‐soluble, biodegradable, biocompatible, non‐immunogenic, highly hygroscopic, and viscoelastic.^8^

HA is the GAG that displays the simplest structure and, unlike conventional GAGs, does not contain sulfate groups and is not covalently linked to core proteins to form proteoglycans. Conversely, it may be free or it can bind indirectly through particular protein bonds (hyaldherins) to proteoglycans to form giant macromolecules, which provide the physical properties of connective tissues.^9^ Furthermore, it is the only GAG not synthesized in the Golgi apparatus but within the plasma membrane.^10^ Specifically, it is synthesized by hyaluronan synthase (HAS), a class of integral membranes of which there are three types in vertebrates (HAS1, HAS2, and HAS3).^11^

HA is the major component of the extracellular matrix (ECM) of soft connective tissues. It is ubiquitous in body tissues such as the skin, synovial fluid, heart valves, umbilical cord, brain, vitreous body, and periodontal tissues.^12^ The average adult human body has 15 g of HA, of which 33% is replaced every day.^13^ In addition to animal and human origin, HA can be extracted from bacteria, for example, the Streptococcus genus.^14^

The biological properties of HA are diverse and exert specific functions depending on the biomolecular characteristics and its chemical–physical status. What does vary is the size, the stoichiometric configuration, and the concentration of the glycosaminoglycan polymer.

**FIGURE 1:**
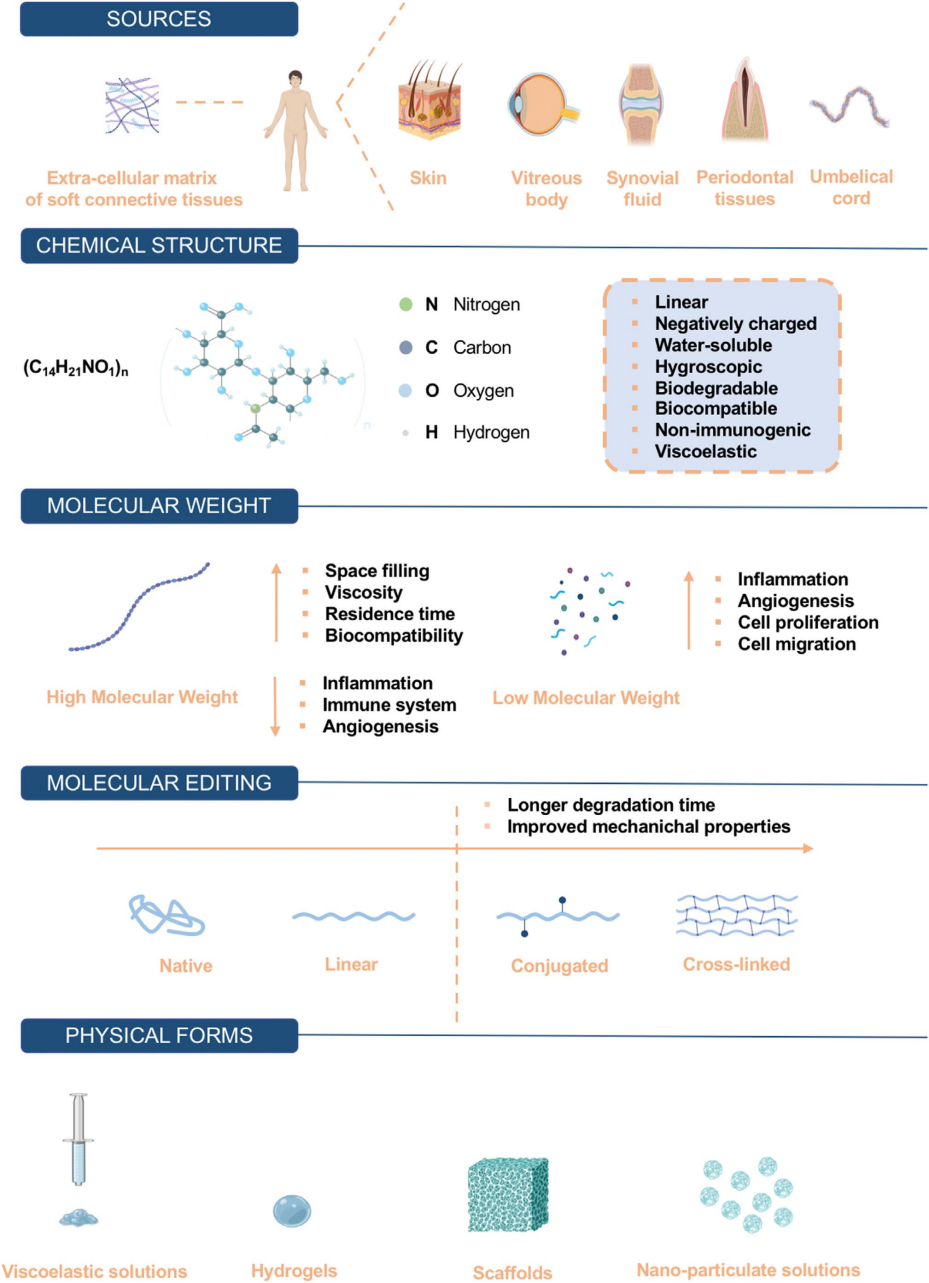
Illustration of the sources, chemical structure, molecular weights, molecular editing, and physical forms of hyaluronic acid. Created in BioRender.

Sources, chemical structure, molecular weights, molecular editing, and physical forms of hyaluronic acid are illustrated in Figure 1.

### 3.1 Molecular weight

The classification of molecular weight (MWs) of HA is not unanimous. In the literature, HA is considered high molecular weight (HMW) when it exceeds 500–1000 kDa.^15^

The length of the linear HA polymer ranges between 10 nm and 25 μm, and the number of repeating disaccharides could reach up to 25 000 disaccharide units.^16^ In its native state, HA from animals has an extremely HMW (up to 20 000 kDa) and varies depending on the tissue type and condition (e.g., inflammation). The HA from microbial pathogens has a molecular weight between 1000 and 4000 kDa.^15^ In vivo, low molecular weight (LMW) HA is produced by the degradation of HA, which could occur through (i) non‐specific degradation mediated by reactive oxygen species (ROS) or (ii) enzymatic processes by hyaluronidase (HYALs).^17^ In industrial manufacturing, LMW‐HA can also be produced by controlled depolymerization of HMW‐HA using physical treatment, irradiation, acid treatment, ozonolysis, and metal catalyzed radical oxidation.^2^

HMW‐HA has antiangiogenic, immunosuppressive, anti‐inflammatory, and space‐filling properties.^8^, ^18^ Furthermore, it exhibits higher viscosity, longer residence time, and higher biocompatibility when compared to LMW‐HA.^19^

In contrast, LMW‐HA has immunostimulatory, inflammatory, and angiogenic properties.^20^, ^21^, ^22^ Furthermore, the degraded HA fragments stimulate cell proliferation, cell migration, and wound healing.^18^

### 3.2 Molecular editing

Native HA is readily degraded by the enzymes, the HYALs, showing poor mechanical properties and a short in vivo residence time. These attributes limit its engineering applicability and its use for longer residence times in the body.^7^ However, HA can be physically or chemically modified to produce a more resistant biomaterial while still maintaining biodegradable and biocompatible properties. In this regard, HA can be considered a desirable polymer due to its linear nature and the availability of functional groups (hydroxyl, carboxyl, and acetyl) which can be chemically edited in two ways: either by conjugation or cross‐linking.^23^ Conjugation involves grafting monofunctional molecules onto a chain of HA via a single covalent bond, while cross‐linking uses polyfunctional compounds to link together several chains of native or conjugated HA via two or more covalent bonds.^24^ Cross‐linked hyaluronan (cHA) can be prepared from native HA (direct cross‐linking) or from its conjugates by synthesis (cross‐linking of functionalized HA).^25^, ^26^

### 3.3 Physical forms

HA is highly versatile due to its easily modifiable chemical structure that enables the creation of various physical forms for biomedical applications, including viscoelastic solutions, hydrogels, electrospun fibers, scaffolds, flexible sheets, and nanoparticle solutions. Each form has a specific function.^27^

## 4 BIOLOGICAL FUNCTIONS

**FIGURE 2:**
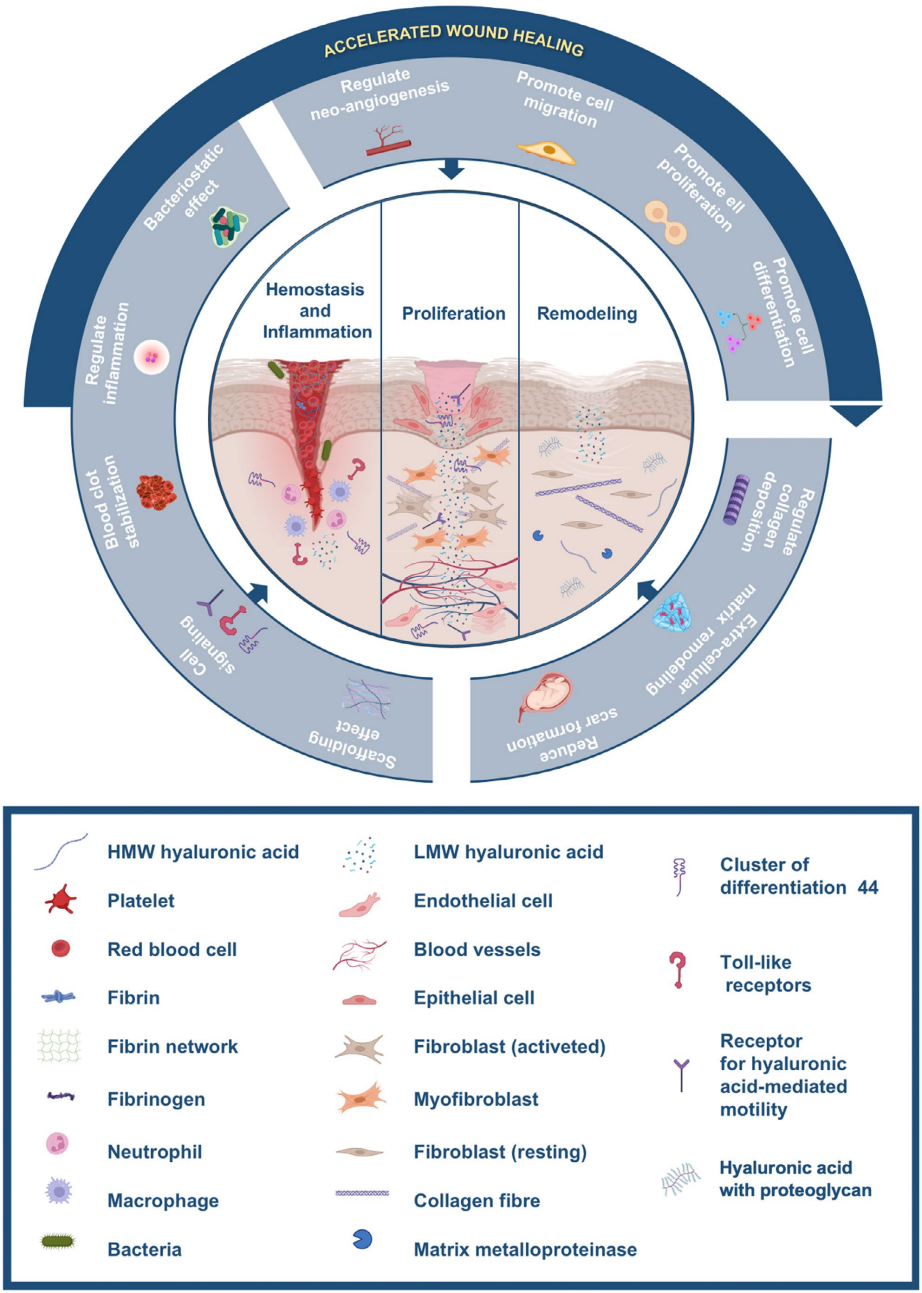
Graphical representation of the biological properties exerted by high molecular weight (HMW) and low molecular weight (LMW) hyaluronic acid during the wound healing process. Created in BioRender.

Previously, HA was simply considered for its role as a passive filler and scaffold, serving as a supporting structure because of its large molecular size. Conversely, nowadays it is conceived as a powerful bioactive molecule capable of exerting functional influences on its surrounding environment. Its role has been shown to be beneficial both in maintaining the structural integrity and homeostasis of ECM and in tissue regeneration and repair. In this section, most of the biological functions of HA are presented based on their sequential role in the wound healing phases of hemostasis and inflammation, proliferation, and remodeling (Figure 2).

### 4.1 Cell signaling

HA facilitates communication between the extracellular matrix and cells. Numerous receptors present in all tissues have the ability to interact with HA. The signaling pathways activated by these receptors can regulate various genes, including chemokines, while also influencing cytoskeleton interactions through cytoplasmic receptor domains. The primary cell surface receptors for HA include cluster of differentiation 44 (CD44), which is the most prevalent receptor involved in HA signaling, receptor for hyaluronic acid‐mediated motility (RHAMM), intercellular adhesion molecule 1 (ICAM‐1), lymphatic vessel endothelial hyaluronan receptor (LYVE‐1), hyaluronic acid receptor for endocytosis (HARE), and toll‐like receptors (TLRs).^28^

### 4.2 Clot stabilization

**FIGURE 3:**
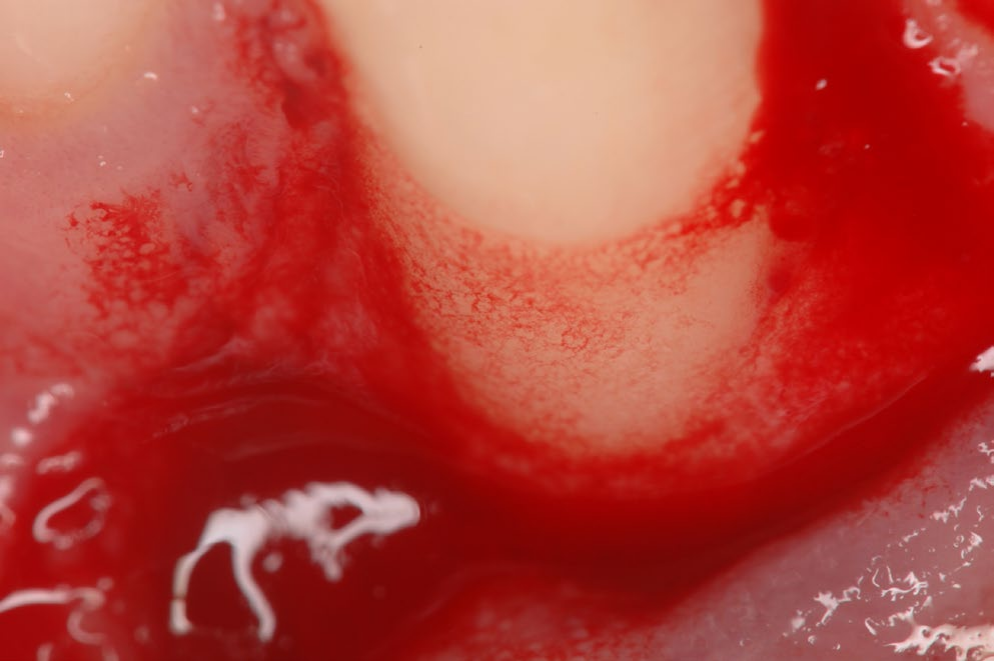
Clinical illustration of clot stabilization after intra‐surgical cHA application on root surface.

Platelets store significant amounts of HMW‐HA in their cytoplasm.^29^ During the initial clot formation, platelets deposit fibrinogen. Fibrinogen, an HA‐binding protein, along with its fibrin product, contributes to maintaining a localized concentration of HA. The HMW‐HA serves as the architectural matrix for the deposition of clotted fibrin.^30^ Figure 3 shows the clot stability once cHA was applied to the root surface intra‐surgically.

### 4.3 Edema formation and increase of vascular permeability

Due to its hygroscopic nature, the initial HMW‐HA can expand its solvent domain up to 10 000 times its original polymer volume. As a result, there is a significant accumulation of HA in the edema fluid, creating space that allows for the infiltration of the first inflammatory cells.^31^, ^32^, ^33^

### 4.4 Anti/pro inflammatory activity

The anti‐inflammatory effects are attributable to interactions of HMW‐HA with CD44.^34^ They are exerted by decreasing interleukin (IL)−1β, IL‐6, tumor necrosis factor‐α (TNF‐α), and prostaglandin E2 (PGE2) production.^35^, ^36^, ^37^

Many of the pro‐inflammatory effects of LMW‐HA are due to its interactions with CD44 and the TLR2 or TLR4.^34^ During the process of wound healing, the initial recruitment of acute inflammatory cells—leukocytes—is facilitated by the binding of HA with CD44 regulating the cells' migration. Increased production of HA by damaged or disrupted endothelial cells could contribute to the HA involved in this recruitment mechanism.^38^ Subsequently, the TLR2 and TLR4 receptors respond to small HA fragments, triggering the expression of inflammatory genes in macrophages. This stimulation leads to the expression of tumor necrosis factor alpha (TNF‐α) and various chemokines, which aid in accelerating the wound healing process through inflammatory mechanisms.^39^

### 4.5 Bacteriostatic effect

Microbial dysbiosis interacts with host genetic factors to influence periodontal disease progression and healing.^40^ HA can exert diverse bacteriostatic effects on certain oral and non‐oral microorganisms in their planktonic phase, depending on its MW and concentration. The highest bacteriostatic effect was observed with high concentrations of medium molecular weight HA (1300 kDa).^41^ A recent in‐vitro study explored the effects of various HAs on the interactions between periodontal biofilms and immune cells. The findings revealed that both high molecular weight HA and cross‐linked high molecular weight HA possess anti‐biofilm properties in the periodontal environment.^42^

### 4.6 Anti/pro angiogenetic activity

HMW‐HA and LMW‐HA show opposite properties regarding angiogenesis. While HMW‐HA suppresses angiogenesis by inhibiting endothelial cell migration and proliferation,^43^ it is promptly cleaved into LMW‐HA fragments once endothelial cells are disrupted.^44^ LMW‐HA enhances angiogenesis to ensure blood supply to the healing area. It also controls the expression of vascular endothelial growth factor (VEGF), and its signaling pathways are mediated by both CD44 and RHAMM receptors.^45^, ^46^, ^47^

### 4.7 Migration, proliferation and differentiation activation

Numerous signaling pathways, involving both cell migration and proliferation, are activated by HA fragments.^18^, ^20^ Initially, cell movement induced by HA is mediated by RHAMM receptors.^48^ Subsequently, keratinocyte proliferation is regulated by the complex interaction between HA and CD44 at the wound edges, while the heparin‐binding form of epidermal growth factor (EGF) contributes to stimulating HA synthesis.^49^, ^50^ In the later stages of healing, fibroblasts, the major players, invade the wound area to deposit collagen. Their invasion and proliferation into the fibrin matrix depend on fibronectin coupled with HA.^51^, ^52^ Fibroblasts themselves are stimulated to produce HA by transforming growth factor beta (TGF‐β) and suppressor of mothers against decapentaplegic (Smad) signaling. As fibroblasts are stimulated to divide, and HA production is upregulated, this constitutes an autocrine, self‐stimulating system.^53^ It is important to mention that despite cell proliferation having been associated with a high level of HA, especially during the initial stages of mitosis, HA itself does not exhibit direct mitogenic activity.^48^

Fibroblasts within the granulation tissue—a complex and dynamic tissue formed along with endothelial cells and inflammatory cells during wound healing—express smooth‐muscle actin and myosin, differentiating into myofibroblasts.^54^ This process is orchestrated by HA present in the granulation tissue, along with CD44. Indeed, HA plays a crucial role in the appearance and maintenance of the myofibroblast phenotype, primarily through TGF‐β1‐dependent steps.^55^, ^56^ Moreover, HA‐induced differentiation has been observed in mesenchymal stem cells (MSCs), embryonic stem cells (ESCs) and neural progenitor cells (NPCs).^57^, ^58^

### 4.8 Early collagen deposition

Following the granulation stage of wound healing, angiogenesis slows down and fibroblasts deposit collagen. Type III collagen, which is soft and malleable, is deposited first, induced by HA. This serves as a temporary patch that does not contribute to the wound's tensile strength.^59^

### 4.9 Tissue remodeling

Under normal circumstances, HA is typically cleared from the wound site by day 10 following injury. At this stage, Type I collagen becomes predominant in the fibrous scar, as Type III collagen is being removed. It has been suggested that the specific degradation of Collagen III may be induced by collagenolytic matrix metalloproteinases (MMPs) in the presence of HA.^60^

### 4.10 Reduced scar formation and fetal‐like wound healing

In hypertrophic scars and keloids, the amount of hHA is progressively decreased compared to normal scars.^61^, ^62^ In contrast, amniotic fluid and embryonic tissues are abundant in HA and exhibit persistent Type III collagen levels. It is widely recognized that fetal wound healing occurs without scar formation until the latter part of the second trimester.^63^, ^64^, ^65^ HA is proposed as a mechanism supporting this scarless repair, also through the upregulation of TGF‐β3.^64^, ^66^

## 5 EFFECTS ON PERIODONTAL TISSUE CELLS: IN VITRO STUDIES

**FIGURE 4:**
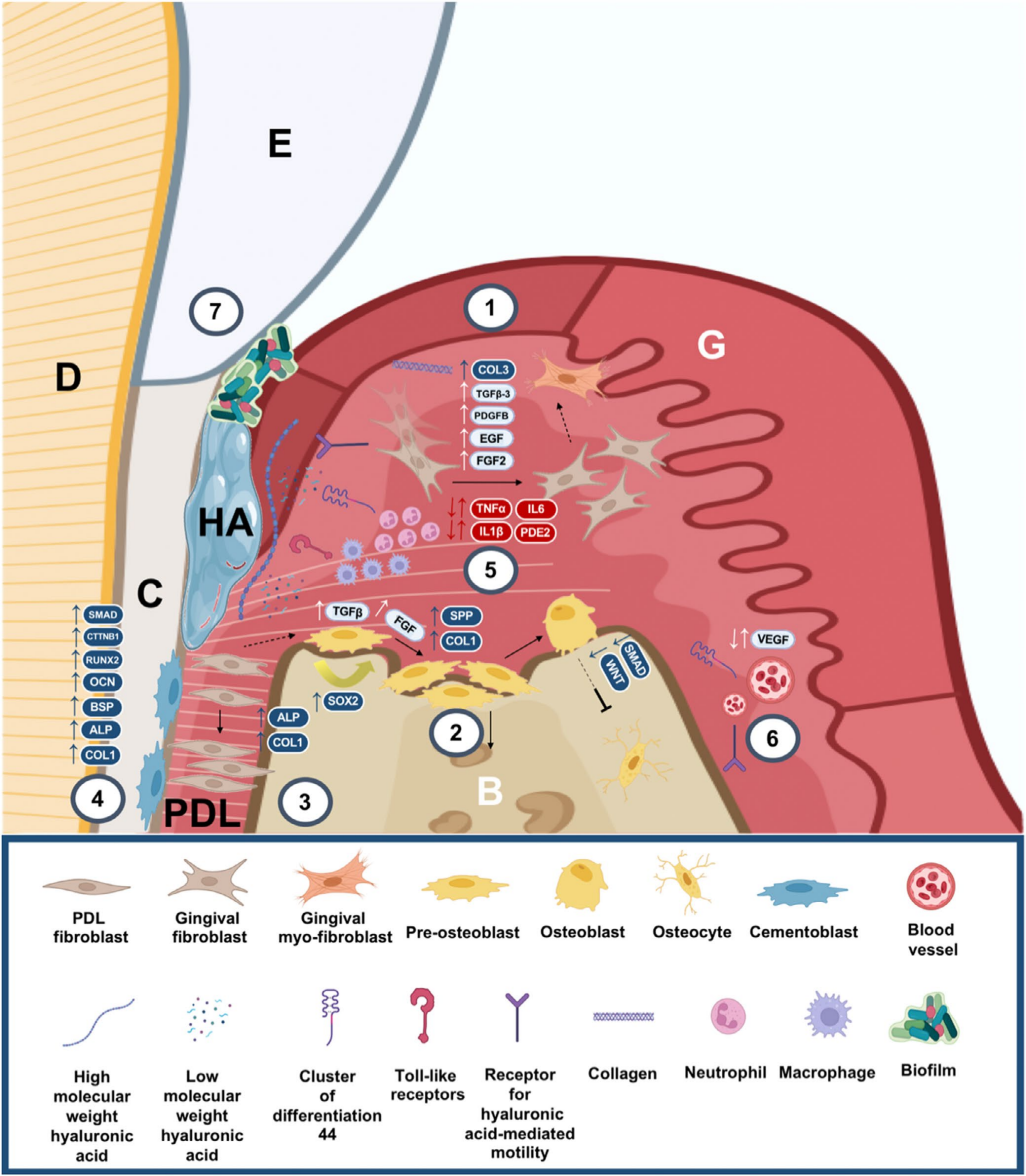
Diagram depicting the behavior of periodontal tissues in the presence of hyaluronic acid (HA). Following HA application, the following effects were observed: (1) increased migration, proliferation, myofibroblast differentiation, Type III collagen deposition, extracellular matrix (ECM) remodeling, and expression of scarless wound healing genes by gingival fibroblasts; (2) increased self‐renewal, proliferation and production of bone matrix, and decreased late osteogenic differentiation of pre‐osteoblasts; (3) improved viability, adhesion, proliferation and early osteogenic differentiation of periodontal ligament cells; (4) increased expression of cementoblast‐specific genes and mineralized tissue markers by cementoblasts (C); (5) regulation of the inflammatory response; (6) regulation of neo‐angiogenesis; (7) bacteriostatic effect. ALP, alkaline phosphatase; B, bone; BSP, bone sialoprotein; C, cementum; COL1, Type 1 collagen; COL3, Type 3 collagen; CTNNB1, β‐catenin; D, dentin; E, enamel; EGF, epidermal growth factor; FGF2, fibroblast growth factor 2; G, gingiva; HA, hyaluronic acid; IL1β, interleukin 1 beta; IL6, interleukin 6; OCN, osteocalcin; PDE2, prostaglandin E2; PDGFB, platelet‐derived growth factor B; PDL, periodontal ligament; RUNX2 runt‐related transcription factor 2; SMAD, suppressor of mothers against decapentaplegic; SOX2, SRY‐Box transcription factor 2; SPP, secreted phosphoprotein; TGFβ, transforming growth factor beta; TGFβ‐3, transforming growth factor beta 3; TNF‐α, tumor necrosis factor‐alfa; VEGF, vascular endothelial growth factor; WNT, Wnt signaling pathways. Created in BioRender.

HA is found in various body fluids including saliva and gingival crevicular fluid, as well as in mineralized and non‐mineralized tissues of the periodontium.^67^, ^68^, ^69^ Higher levels of HA are detected in the gingiva and periodontal ligament compared to cementum and alveolar bone.^70^, ^71^, ^72^, ^73^ In this section, the effects of HA on periodontal tissue cells are presented based on in vitro studies. Figure 4 shows a diagram depicting the behavior of periodontal tissues in the presence of hyaluronic acid.

### 5.1 Bone cells

A study demonstrated that LMW‐HA maximizes the osteogenic potential of mesenchymal cells by promoting the subsequent differentiation and proliferation of osteoprogenitor cells, facilitated by its open spatial organization. This led to an improved formation of bone colonies in vitro.^74^

More recently, a study showed that two different HMW‐HAs greatly enhanced osteoprogenitor growth and bone matrix protein expression. Upon HA treatment, there was increased expression of genes encoding TGF‐β1 and fibroblast growth factor‐1 (FGF‐1), along with enhanced phosphorylation of downstream signaling molecules Smad‐2 and extracellular signal‐regulated kinase 1/2 (Erk1/2). HA treatment led to significant upregulation of the transcription factor Sox2 and its direct transcription targets, including critical stemness genes such as the yes‐associated protein 1 and the polycomb complex protein 1. Active β‐catenin levels decreased in HA‐treated cells due to phosphorylation and subsequent degradation. Conversely, HA inhibited the expression of late osteogenic markers and bone morphogenetic protein (BMP). Furthermore, Wnt signaling pathway targets were downregulated, while pathway inhibitors were upregulated. These effects resulted in inhibited progression of the osteogenic differentiation in osteocytes. In conclusion, HA strongly induces osteoprogenitor growth and maintains stemness, potentially regulating the balance between self‐renewal and differentiation during bone regeneration following reconstructive procedures.^75^

### 5.2 Cementum cells

A study examined the effects of various dilutions (0, 1:2, 1:4, 1:8, 1:16, 1:32, 1:64, 1:128) of HA on cell viability, migration, mineralization, and gene expressions of cementoblasts. The results indicated increased numbers of mineralized nodules, along with a significant improvement in mRNA expressions of mineralized tissue markers including collagen type I (COL‐1), bone sialoprotein (BSP), osteocalcin (OCN), runt‐related transcription factor 2 (Runx2), and alkaline phosphatase (ALP) with HA treatments.^76^ Conversely, the balance and function of such critical extracellular matrix components, including BSP, Type I collagen, and Type III collagen are disrupted during periodontitis, leading to impaired tissue regeneration and progressive periodontal destruction.^77^ Additionally, the study showed that mRNA expressions of Smad‐2, ‐3, ‐7, and β‐catenin (Ctnnb1) were upregulated. Overall, the study demonstrated that HA affected the expression of both mineralized tissue markers and cementoblast‐specific genes, suggesting its potential role in cementum regeneration.^76^

### 5.3 Periodontal ligament cells

Both non‐cross‐linked and cross‐linked HA (cHA) showed high viability of periodontal ligament (PDL) cells and promoted cell proliferation. Furthermore, early osteogenic differentiation was observed when osteogenic differentiation medium was added to the standard cell growth media, as evidenced by the increased expression of COL1 and ALP.^78^ Accordingly, Takeda et al.^79^ showed that HMW‐HA enhanced PDL cell adhesion and proliferation.

In a recent study, the effects of cHA, human serum (HS) and their combination on PDL cells and periodontal biofilm were investigated. The study found that cHA alone or in combination slightly decreased the colony‐forming unit counts and reduced the metabolic activity of biofilm. Furthermore, these substances did not negatively affect the adhesion of PDL fibroblasts to dentin.^80^

### 5.4 Gingival and palatal fibroblasts

Asparuhova et al.^81^ demonstrated that two formulations of HMW‐HA were fully biocompatible and did not adversely affect the viability of human palatal fibroblasts (HPFs) and human gingival fibroblasts (HGFs). They enhanced the proliferative and migratory abilities and induced the expression of collagen type alpha 1 (COL3A1) and TGFβ‐3 genes, characteristic of scarless fetal wound healing. Additionally, these formulations upregulated the expression of genes encoding the platelet‐derived growth factor B (PDGFB), fibroblast growth factor 2 (FGF‐2), and EGF, which play crucial roles in the healing process. Furthermore, HA modulated matrix metalloproteinases gene expression, directly (MMP1 and 8) or indirectly (MMP2 and 3), affecting ECM remodeling. Finally, it has been suggested that protein kinase B (Akt), Erk1/2, and p38 mitogen‐activated protein kinase could serve as the signaling molecules through which HA exerts its effects on oral fibroblasts.

## 6 ANIMAL HISTOLOGICAL EVIDENCE OF PERIODONTAL REGENERATION

Theoretically, a human block biopsy is the only method that can demonstrate periodontal regeneration.^82^ However, this approach has several drawbacks, including a lack of control treatments, the use of teeth with low regenerative potential, and obvious ethical issues.^82^, ^83^ Thus, given the scope for the ethical use of experimental animals, histological outcomes including quantitative analysis using various histomorphometric parameters and qualitative analysis have been extensively reported in pre‐clinical studies.^84^ In this section, the effects of various cHA applications on periodontal wound healing/regeneration in experimental animals are presented.

### 6.1 Surgical therapy for gingival recession defects

**FIGURE 5:**
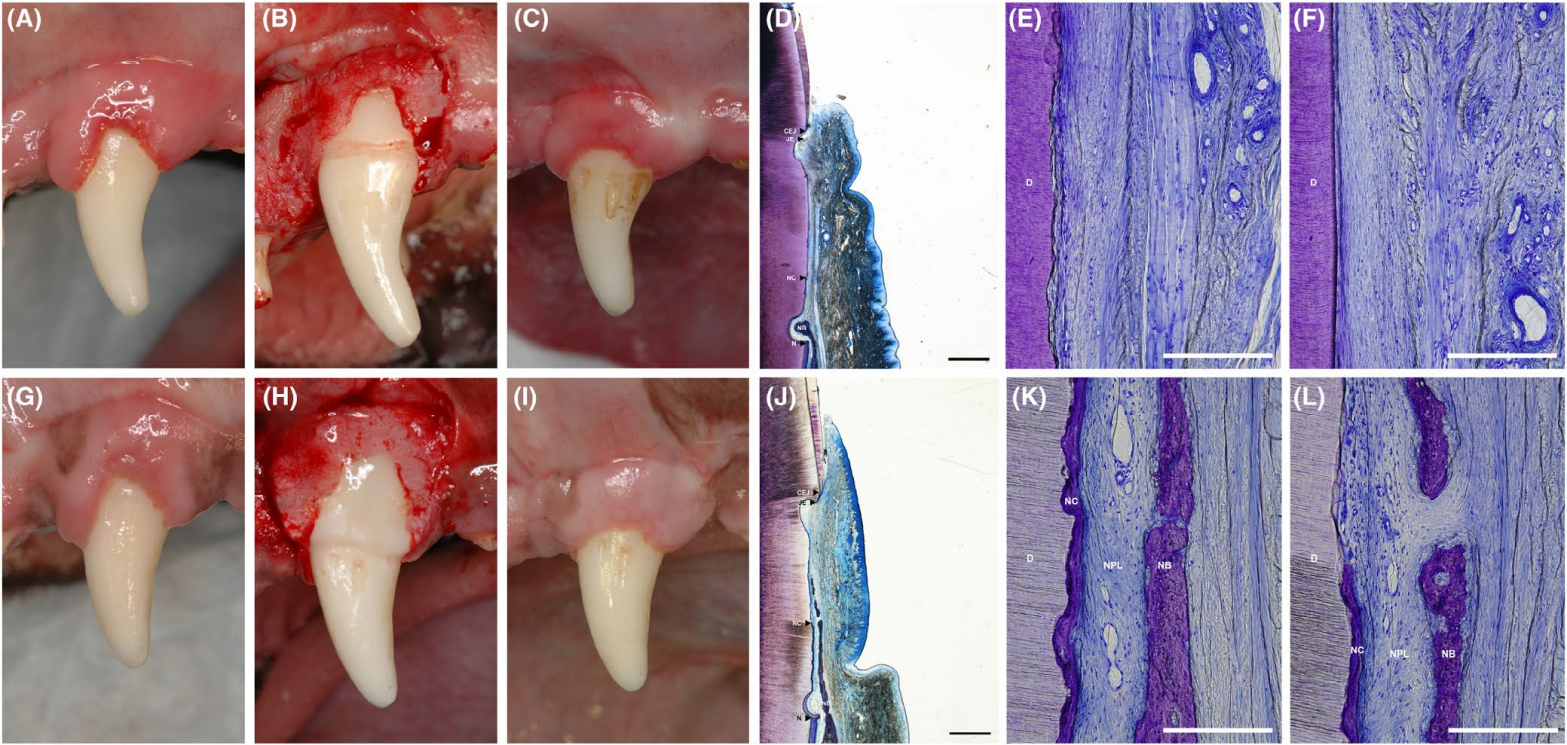
Clinical photographs and representative histological photomicrographs of buccal gingival recession defects. CAF group (A) at baseline. (B) Defect on the root after debridement. (C) after 10 weeks of healing. (D) Overview. (scale bar, 1 mm, toluidine blue staining). (E) Higher magnification of the middle portion of the defect treated by CAF. (scale bar, 200 μm, toluidine blue staining). (F) Higher magnification of the coronal portion of the defect treated by CAF. (scale bar, 200 μm, toluidine blue staining; original magnification 20×) CAF with cHA group (G) at baseline. (H) cHA was applied onto the denuded root surface and (I) after 10 weeks of healing. (J) Overview. (scale bar, 1 mm, toluidine blue staining). (K) Higher magnification of the middle portion of the defect treated with CAF/cHA. (scale bar, 200 μm, toluidine blue staining) (L) Higher magnification of the coronal portion of the defect treated with CAF/cHA. (scale bar, 200 μm, toluidine blue staining) CEJ, cemento‐enamel junction; D, root dentin; JE, apical end of junctional epithelium; N, apical end of apical notch; NB, new bone; NC, new cementum; NPL, newly formed periodontal ligament. Adapted from Shirakata et al.^85^

The clinical and histological healing of gingival recession defects on bilateral upper canines treated using a coronally advanced flap (CAF) with or without cHA gel (Hyadent BG®, REGEDENT AG, Zurich, Switzerland) in dogs was first evaluated by Shirakata et al.^85^ (Figure 5). At 10 weeks, both CAF and CAF/cHA treatment groups showed statistically significant post‐surgical improvements in terms of gingival recession (GR) when compared with baseline (CAF group; *p* < 0.01, CAF/cHA group; *p* < 0.001). In the CAF/cHA group, there was a statistically significant difference (reduction) in the probing pocket depth (PPD) compared with baseline (*p* < 0.05). Statistically significant differences were found in clinical attachment level (CAL) (*p* < 0.05) and the width of gingival recession (GR) (*p* < 0.01) favoring the CAF/cHA group (Figure 5C,E). Minimal new bone formation was observed in the CAF group (Figure 4D). No formation of new connective tissue attachment (i.e., new cementum with inserting collagen fibers) was observed, and connective tissue fibers were aligned parallel to the root surface (Figure 5E,F). In the CAF/cHA group, bone formation was noted extending from the apical notches toward the coronal region of the defects (Figure 5J–L). In these defects, dense collagen fibers were seen inserting into the newly formed cementum, obliquely oriented to the root surface (Figure 5K,L). The highly vascularized new periodontal ligament‐like tissue, which was formed between the new cementum and new bone, maintained its width up to the coronal portion (Figure 5K,L). Bone formation was statistically significantly greater in the CAF/cHA group than in the CAF group (*p* < 0.05). Formation of cementum and connective tissue attachment was statistically significantly higher in the CAF/cHA group compared with the CAF group (*p* < 0.05). These results indicated that the combination of cHA and CAF promoted periodontal wound regeneration in gingival recession defects.

### 6.2 Surgical therapy for intrabony defects

**FIGURE 6:**
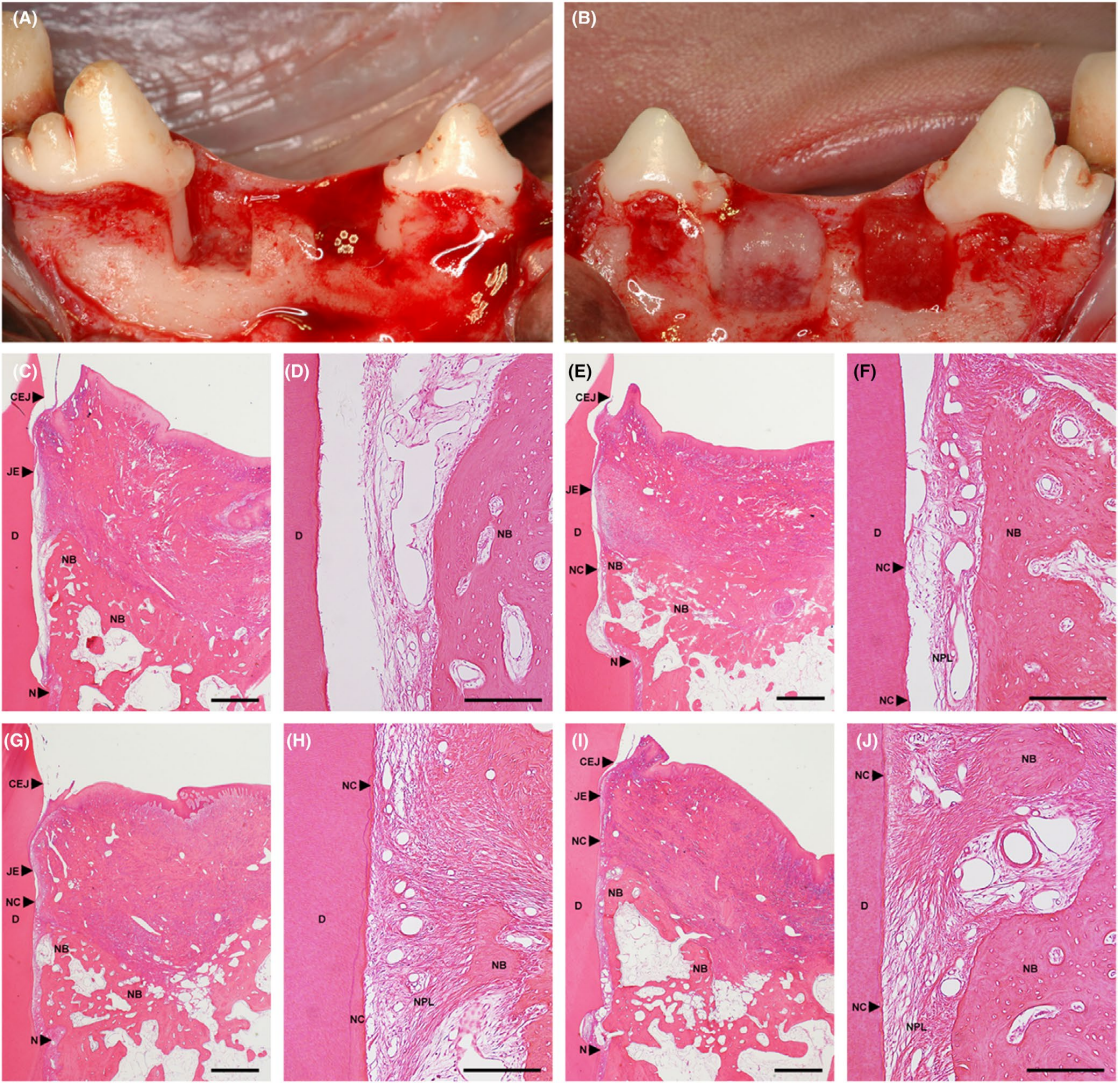
Clinical photographs showing the surgically created and treated two‐wall intrabony defects. (A) Left: open flap debridement (OFD), right: cross‐linked hyaluronic acid (cHA) gel application, (B) Left: cHA gel and collagen matrix (CM) construct (cHA/CM) was placed into the defect, right: CM placement. Representative histological photographs of a two‐wall intrabony defect treated with OFD. (C) Overview. (scale bar, 1 mm, hematoxylin and eosin staining). (D) Higher magnification of the coronal portion of the bone crest. (scale bar, 200 μm, hematoxylin and eosin staining) Representative histological photographs of a two‐wall intrabony defect treated with OFD and CM. (E) Overview. (scale bar, 1 mm, hematoxylin and eosin staining). (F) Higher magnification of the coronal portion of the bone crest. (scale bar, 200 μm, hematoxylin and eosin staining) Representative histological photographs of a two‐wall intrabony defect treated with OFD and cHA. (G) Overview. (scale bar, 1 mm, hematoxylin and eosin staining). (H) Higher magnification of the coronal portion of the bone crest. (scale bar, 200 μm, hematoxylin and eosin staining) Representative histological photographs of a two‐wall intrabony defect treated with OFD, cHA, and CM implantation. (I) Overview. (scale bar, 1 mm, hematoxylin and eosin staining). (J) Higher magnification of the coronal portion of the bone crest. (scale bar, 200 μm, hematoxylin and eosin staining) CEJ, cemento‐enamel junction; D, root dentin; JE, apical end of junctional epithelium; N, apical end of apical notch; NB, new bone; NC, new cementum; NPL, newly formed periodontal ligament. Adapted with permission from Quintessence International Publishing from Shirakata et al.^86^

Later, the effects of cHA with or without a cross‐linked porcine collagen matrix (CM: Fibro‐Gide®, Geistlich Pharma, Wolhusen, Switzerland) on periodontal wound healing and regeneration of non‐contained two‐wall intrabony defects in dogs were evaluated.^86^ The surgically created defects received one of the following treatments: open flap debridement (OFD group), OFD and grafting of CM (CM group), OFD and cHA (cHA group), and OFD and a combination of cHA and CM (cHA/CM group) (Figure 6A,B). Generally, clinical healing was successful and no complications were observed for any of the defects. The cHA/CM treatment group was superior to the other groups in terms of new bone formation. However, no significant differences were apparent among the treatments (Figure 6C,E,G,I), possibly due to spontaneous bone healing occurring in this acute type of intrabony defect. In the OFD and CM groups, no or only a small amount of new cementum was observed, with sparse collagen fibers detached from or parallel to the root surfaces (Figure 6C–F). Conversely, new acellular or cellular cementum with inserting collagen fibers running perpendicular to the root surfaces was observed in the cHA and cHA/CM treatment groups. Additionally, highly vascularized and dense new periodontal ligament‐like tissue had formed between new cementum and new bone, maintaining its width up to the coronal portion in the cHA‐applied treatment groups (Figure 6G–J). Histomorphometric analysis revealed tendencies toward greater formation of new cementum and new bone in the cHA‐applied groups compared with the OFD and CM groups, although the differences were not significant. However, new attachment length (*p* < 0.05) and periodontal ligament score (*p* < 0.01) were significantly greater in the cHA and cHA/CM groups when compared with the OFD group.

### 6.3 Surgical therapy for furcation defects

**FIGURE 7:**
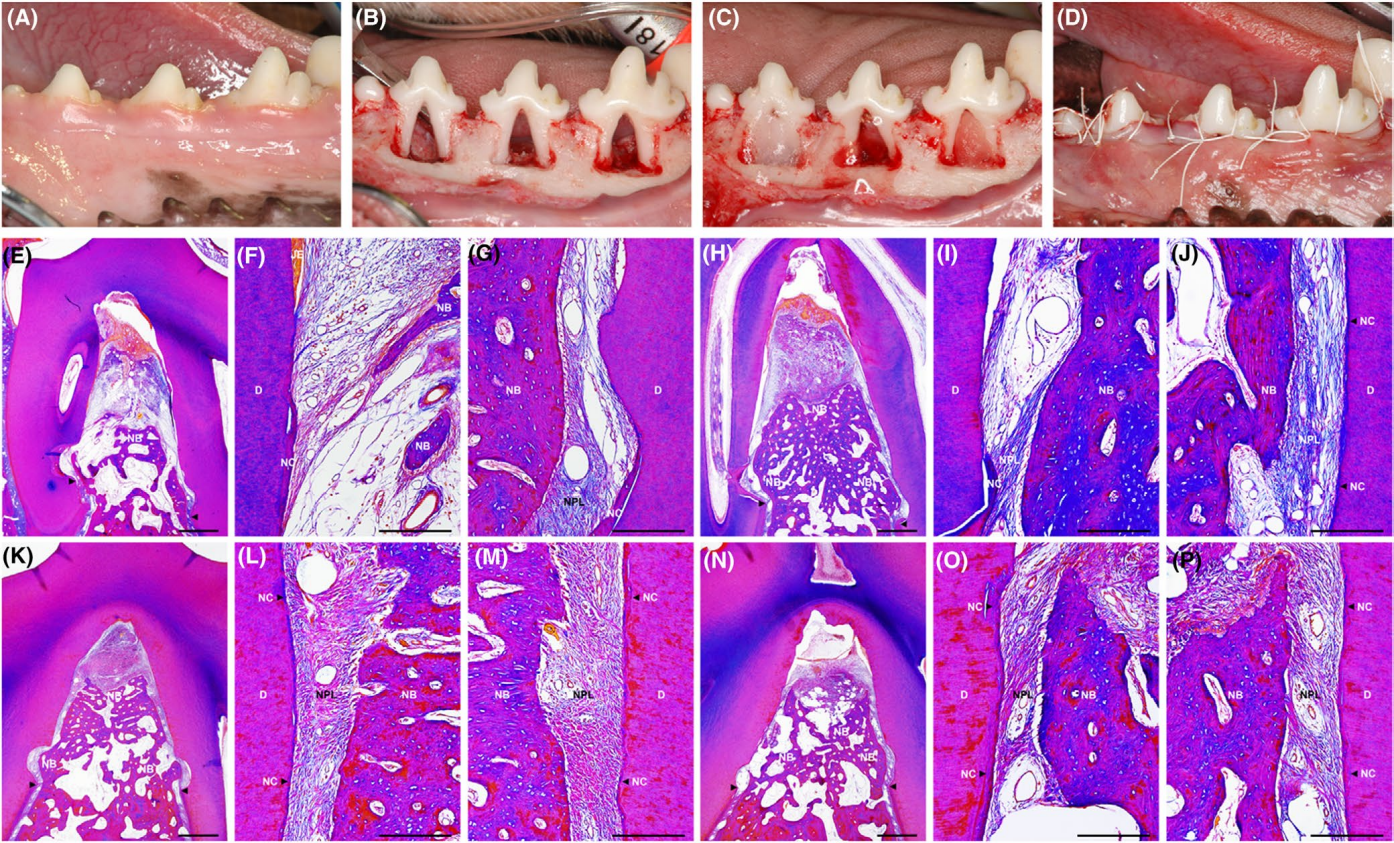
Clinical illustration of the treatment procedure. (A) Prior to reconstructive surgery. (B) Surgically created Class III furcation defects. (C) The defects received cHA with CM, cHA and CM (left to right). (D) Flap repositioning and suturing. Representative histological photographs of Class III furcation defects in different groups. OFD group. (E) Overview. (scale bar, 1 mm, azan staining). Arrowhead. Notch (apical extension of root planing). (F) Higher magnification of the framed area (left) in (E) (scale bar, 200 μm, azan staining) (G) Higher magnification of the framed area (right) in (E) (scale bar, 200 μm, azan staining). CM group. (H) Overview. (scale bar, 1 mm, azan staining). Arrowhead. Notch (apical extension of root planing). (I) Higher magnification of the framed area (left) in (H) (scale bar, 200 μm, azan staining) (J) Higher magnification of the framed area (right) in (H) (scale bar, 200 μm, azan staining). cHA group. (K) Overview. (scale bar, 1 mm, azan staining). Arrowhead. Notch (apical extension of root planing). (L) Higher magnification of the framed area (left) in (K) (scale bar, 200 μm, azan staining) (M) Higher magnification of the framed area (right) in (K) (scale bar, 200 μm, azan staining). cHA with CM group. (N) Overview. (scale bar, 1 mm, azan staining). Arrowhead. Notch (apical extension of root planing). (O) Higher magnification of the framed area (left) in (N) (scale bar, 200 μm, azan staining). (P) Higher magnification of the framed area (right) in (N) (scale bar, 200 μm, azan staining). D, root dentin; JE, apical end of junctional epithelium; N, apical end of apical notch; NB, new bone; NC, new cementum; NPL, newly formed periodontal ligament Adapted from Shirakata et al.^87^

Subsequently, the effects of cHA with or without CM were histologically evaluated in the treatment of Class III furcation defects in dogs.^87^ The Class III furcation defects created in canine mandibular premolars received one of four treatments from the previous study, namely OFD, OFD with CM, OFD with cHA, or OFD with cHA and CM (Figure 7A–D). Postoperative clinical healing was again uneventful at all sites. After 10 weeks, bone formation was consistently observed in the cHA and cHA/CM groups compared with the OFD and CM groups. However, no complete furcation closure was observed in any of the four treatment modalities (Figure 7E,H,K,N). In the OFD and CM groups, new cementum was confined to the apical parts of the defects with apical migration of the junctional epithelium, indicating rather reparative‐type periodontal healing (Figure 7F,G,I,J). In contrast, dense, functionally oriented Sharpey's fibers were again clearly observable between the new bone and cementum in the cHA and cHA/CM groups (Figure 7L,M,O,P). No statistically significant differences were detected among the treatment groups in terms of area measurements, although connective tissue areas were smaller and new bone areas were larger in the cHA‐applied groups compared with the OFD and CM treatment groups. Interestingly, again, the results from linear measurements showed statistically significantly greater amounts of new attachment and new cementum formation in the groups that received cHA when compared with the OFD group (*p* < 0.05).

### 6.4 Novel nonsurgical therapy for experimental periodontitis

**FIGURE 8:**
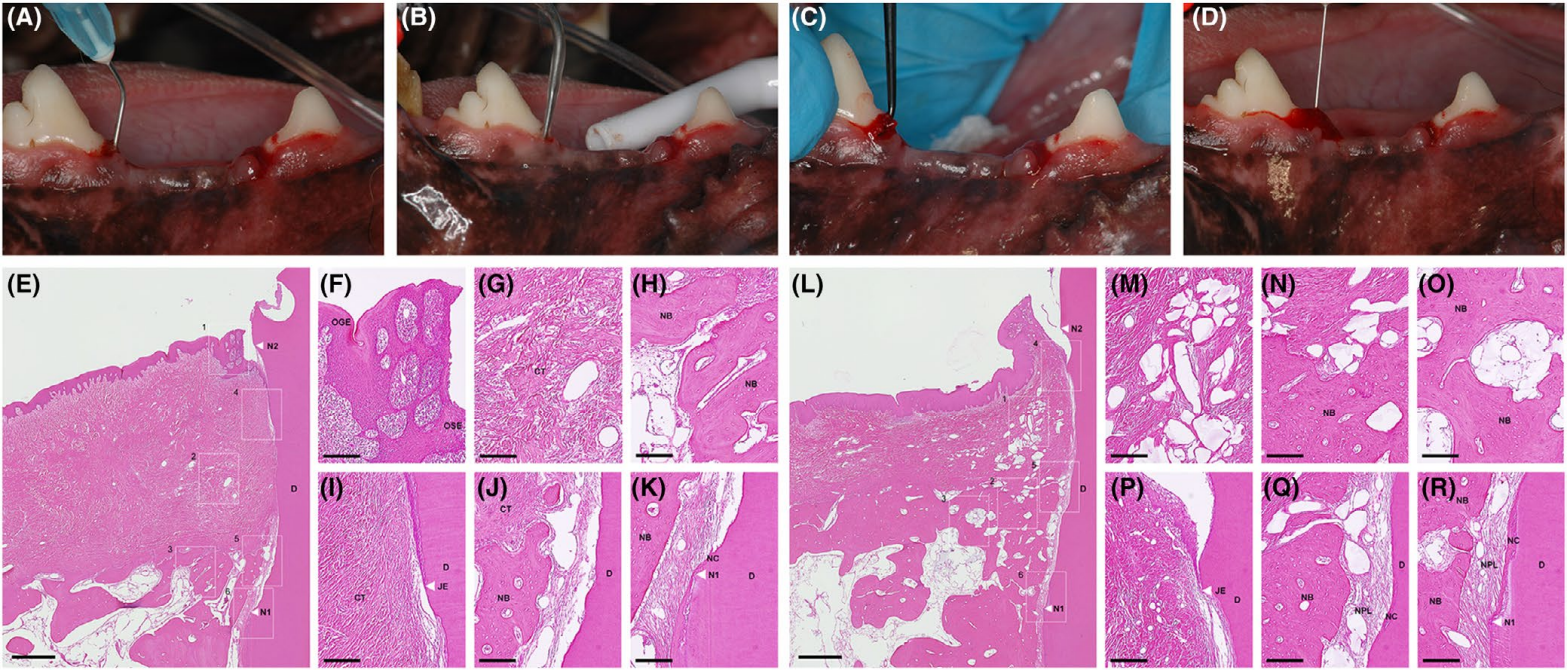
Clinical overview of the treatment procedure. (A) Application of sodium hypochlorite/amino acid gel to the periodontal pocket. SRP performed using an ultrasonic device with an ultrasonic tip (B) and hand instruments (C). (D) Application of a cHA gel to the periodontal pocket. Representative histological photographs of defect treated with SRP alone (control group). (E) Overview. (scale bar, 1 mm; hematoxylin and eosin stain). (F) Higher magnification of the box 1 area. (G) Higher magnification of the Box 2 area. (H) Higher magnification of the Box 3 area. (I) Higher magnification of the Box 4 area. (J) Higher magnification of the Box 5 area. (K) Higher magnification of the Box 6 area. (scale bar, 200 μm; hematoxylin and eosin stain). Representative histological photographs of defect treated with SRP with a sodium hypochlorite and amino acids gel and a cHA gel (test group). (L) Overview (scale bar, 1 mm; hematoxylin and eosin stain). (M) Higher magnification of the Box 1 area. (N) Higher magnification of the Box 2 area. (O) Higher magnification of the Box 3 area. (P) Higher magnification of the Box 4 area. (Q) Higher magnification of the Box 5 area. (R) Higher magnification of the Box 6 area. (scale bar, 200 μm; hematoxylin and eosin stain). CT, gingival connective tissue; D, root dentin; JE, apical end of junctional epithelium; N_1_, apical end of root planing; N_2_, cemento–enamel junction; NB, new bone; NC, new cementum; NPL, new periodontal ligament; OGE, oral gingival epithelium; OSE, oral sulcular epithelium. Adapted from Shirakata et al.^91^

Very recently, a novel two‐step approach consisting of enhanced biofilm removal during non‐surgical therapy by means of a sodium hypochlorite/amino acid followed by application of a cHA gel was suggested to improve the clinical outcomes of nonsurgical periodontal therapy.^88^, ^89^, ^90^ Therefore, Shirakata et al.^91^ assessed the periodontal wound healing following scaling and root planing (SRP) in conjunction with the application of sodium hypochlorite/amino acids and cHA gels in experimental periodontitis in dogs (Figure 8A–D). At 6 weeks after treatment, the test group showed statistically significant improvements in terms of PPD reduction (4.25 ± 0.50 mm and 3.34 ± 0.54 mm, respectively), CAL gain (3.90 ± 0.82 mm and 2.78 ± 0.79 mm, respectively), and BoP reduction (12.5% vs. 75%, respectively) compared to the control (SRP alone) group. In the control group, the slight to moderate widespread inflammatory cell infiltrate was observed at the tip of the gingiva (Figure 8E,F). Periodontal defects were mostly occupied by fibrous connective tissue, and new bone and new cementum formation was limited to the bottom of the defect (Figure 8E,G,H). Most sections in the control group showed non‐functional disordered periodontal ligament‐like tissue or collagen fibers detached from the root surfaces (Figure 8I–K). In the test group, residual cHA with reticular appearance was well integrated with gingival connective tissue in all sites (Figure 8F,L). Some remnants of cHA were observed around/in the newly formed bone and occasionally between new cellular/acellular cementum and new bone (Figure 8L–O,Q,R). Apical extension of the junctional epithelium was mostly restricted at the CEJ (Figure 8P). New bone formation extended from the host bone toward the coronal region of the defect (Figure 8L). A continuous layer of new cementum was seen, with or without inserting collagen fibers running perpendicular to the root surfaces, and was observed covering half of the defect area in most sites (Figure 8L,Q,R). Histomorphometrically, the test group exhibited statistically significant greater formation of new bone, new attachment, and new cementum compared with the control group (*p* < 0.05) at 8 weeks. Thus, adjunctive application of cHA and chloramine gels for non‐surgical treatment may not only yield favorable clinical results but also true periodontal regeneration.

In conclusion, these findings following cHA application in experimental animals have provided histologic evidence for periodontal regeneration to support the clinical use of cHA in various non‐surgical and surgical periodontal therapies. However, further clinical studies are required to develop novel strategies that include the use of cHA combined with appropriate biomaterials (e.g., carriers, scaffolds) for promoting periodontal wound regeneration in non‐contained periodontal defects.

## 7 CLINICAL APPLICATIONS OF HA IN PERIODONTAL THERAPY

HA has appeared as a resourceful tool in the field of dentistry. It has captured significant attention due to its diverse clinical applications and promising outcomes. In periodontal treatment, HA has shown efficacy in promoting tissue regeneration and wound healing. In mucogingival therapy, HA has been utilized to address various soft tissue deficiencies and gingival recession defects. Clinical research on HA underscored its efficacy and safety in periodontal applications. Studies have consistently shown positive outcomes, ranging from improved wound healing to enhanced tissue regeneration. In this section, we briefly summarize clinical research on HA over the past two decades.

### 7.1 Effects on early wound healing

**FIGURE 9:**
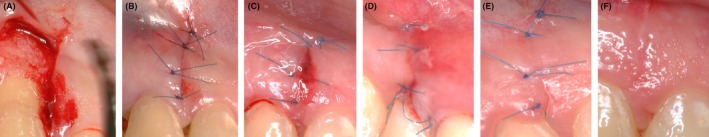
Clinical illustration of early healing at different time intervals at a vertical release incision site. (A) intraoperative HA application; (B) suture; (C) 1 day after surgery; (D) 3 days after surgery; (E) 1 week after surgery; (F) 2 weeks after surgery.

Some studies have attempted to evaluate the early wound healing characteristics of HA in a clinical setting. In an examiner‐masked, randomized, controlled clinical trial, 36 patients requiring a free gingival graft surgery were assigned to three groups.^92^ After palatal graft harvesting, test groups 1 and 2 received 0.2% and 0.8% HA gels, respectively, and the donor sites were protected with a periodontal dressing. The control group was covered only with the periodontal dressing. On Days 3, 7, 14, 21, and 42, complete epithelization (CE), color match, pain, and burning sensation were recorded. It was found that the topical application of HA accelerated palatal wound healing, as determined by a higher proportion of CE and superior color match scores at 21 days, as well as having a positive impact on postoperative pain and burning sensation, specifically during the first week. Canciani et al.^93^ also investigated the effects of the application of an HA gel charged with amino acids on the healing of the gingival tissue at the level of the lower third molar post‐extraction socket. In this split‐mouth randomized controlled clinical and histological study, 10 healthy patients each contributed one test (who received HA three times daily for 10 consecutive days) and one control site (who did not receive HA). Gingival tissue was collected at the time of extraction (T0) and 10 days after extraction (T1) to be analyzed histologically and immunohistochemically. This study showed that the application of the HA gel provided beneficial effects on gingival wound healing in terms of increased microvascular density and improved collagen fiber organization. Notably, a more recent study by Pilloni et al.^94^ has contributed valuable insights into this field, examining both clinical, histological, immunohistochemical, and biomolecular aspects. Using a randomized, split‐mouth, double‐blind study design, gingival biopsies were obtained from 8 patients 24 hours after periodontal surgery at the level of two vertical releasing incisions. The choice of this very short observation period was based on the cellular response that begins immediately after injury, and previous studies have shown that important transcriptional changes occur in the first 12–24 h.^95^, ^96^, ^97^ At the end of the surgical procedure, one vertical releasing incision received HMW HA application (test site), and the other received no treatment (control site). Clinical responses were assessed using the Early Wound Healing Score (EHS). Microvascular density (MVD) and collagen content were assessed by Sirius Red and Masson trichrome staining, and cell proliferation by Ki‐67 immunohistochemistry. To evaluate collagen turnover, the levels of matrix metalloproteinases‐1, matrix metalloproteinases‐2, matrix metalloproteinases‐9, TGF‐β1 proteins and the expression of LOX, MMP1, TIMP1, TGF β1 genes were analyzed by Western blot and real‐time polymerase chain reaction. In contrast to the previously mentioned study, no statistically significant difference was found between test and control sites in terms of the growth of new blood vessels in the early phase of gingival wound healing. Furthermore, increased cell proliferation and a difference in collagen content were not observed. However, HA appeared to enhance extracellular matrix remodeling and collagen maturation, as demonstrated by upregulation of LOX mRNA, matrix metalloproteinases‐1 protein, and TIMP1 gene expression. The latter justified the improvement of clinical parameters at the test sites, as HA could enhance early wound healing. Early periodontal soft tissue healing after using HA is presented in Figure 9.

### 7.2 Clinical outcomes following non‐surgical periodontal therapy

There has recently been great interest in the use of HA gel as monotherapy or as an adjunct to nonsurgical therapy administered as part of initial treatment of periodontitis or repeated instrumentation of residual pockets. In 2019, Eliezer et al.^98^ published a systematic review aimed at evaluating the potential added benefit of the application of HA on the clinical outcomes following non‐surgical or surgical periodontal therapy. Eleven articles reported data on the effect of HA in non‐surgical periodontal therapy in patients affected by periodontitis. In particular, 9 studies showed CAL gain for sites treated with scaling and root planing after 3 months, with a weighted mean difference of 0.73 mm (95% CI 0.28–1.17 mm; *p* < 0.0001), favoring the addition of HA. Eight studies reported data on PD reduction for sites treated with the use of HA versus a control group where no HA was used. The weighted mean difference was −0.36 mm (95% CI −0.54 to −0.19 mm; *p* < 0.0001), favoring the treatment with HA. However, considerable heterogeneity was identified among studies (chi‐squared test *p* < 0.0001). Furthermore, 5 studies showed data on BOP reduction of sites treated with HA versus a control group, with a weighted mean difference of −15% (95% CI −22 to −8%; *p* < 0.001), favoring the experimental treatment.

In the same year, a split‐mouth RCT not included in the previous systematic review reported data from a single topical application of 0.8% HA (Gengigel®, Ricerfarma, Italy) as a coadjuvant to scaling and root planing in periodontal patients.^99^ Results at 3 months of 16 patients showed that, even in the presence of a significant difference in terms of BoP between groups (9.4 ± 4.0 vs. 14.9 ± 8.9, *p* < 0.05), PPD and CAL showed a slight improvement in comparison with the control sides, but the difference was not statistically significant (*p* > 0.05).

The same 0.8% HA gel was tested in another randomized split‐mouth design study on 24 patients with moderate to severe periodontitis evaluated after full mouth SRP.^100^ Plaque index, gingival index, papillary bleeding index, and periodontal probing depths showed statistically significantly higher reductions in test sites than control sites at 6 and 12 weeks. However, CAL did not result in a statistically significant difference between test sites and control sites at 6 or 12 weeks. In addition, the expression of human beta defensin‐2 in the test sites was statistically significantly higher than in the control sites at 6 and 12 weeks.

In 2021, a multi‐center study investigated the additional effect of HA gel in treating residual periodontal pockets over 12 months.^101^ One hundred forty‐four patients with at least one residual periodontal pocket of 5–9 mm in depth in the anterior area were recruited from six university centers. Participants were randomly assigned to subgingival instrumentation with either HA gel (test group) or a placebo (control group) at baseline and after 3 months. PD, BoP, PI, REC, CAL, and microbiological samples for total bacterial count (TBC) and specific bacteria (*Porphyromonas gingivalis*, *Treponema denticola*, *Tannerella forsythia, Fusobacterium nucleatum*) were measured at baseline and every 3 months until the study ended. PD was the primary outcome variable. Both treatments led to statistically significant clinical and microbiological improvements from baseline. Although HA gel showed a tendency for better results, the differences between the test and control groups were not statistically significant.

A different result was reported by Vajawat et al.^102^ using the same HA gel in a split‐mouth RCT in smoker and non‐smoker populations. In fact, statistically significant PPD reduction and CAL gain were observed in both smokers and non‐smokers 3 months after treatment. The improvements in the test sites were statistically significant when compared with those of control sites. In addition, the microbiological analysis showed a statistically significant reduction in *Aggregatibacter actinomycetemcomitans* and *Porphyromonas gingivalis* at the test sites when compared to the controls in both groups.

Ariel et al.^103^ treated residual pockets in 34 subjects affected by Stage 3 periodontitis with a thermosensitive gel formulation containing 0.8% oligo‐HA combined with a preservation system of octenidine HCl 0.625% and phenoxyethanol (POCKET‐X® gel – Prudentix 7110604 Lod, Israel) to scaling and root planning. The test sites were randomly compared with sites treated with SRP alone. Comparisons between the test and control groups revealed that SRP + gel yielded statistically significantly higher PD reductions compared to SRP alone (*p* < 0.0001). Compared to baseline, the test group presented statistically significantly higher CAL gains and BOP reductions than the control group (*p* < 0.0001).

In 2023, Pilloni and coworkers tested the clinical efficacy of a gel containing polynucleotides and HA (REGENFAST, Mastelli S.r.l., Sanremo, Italy) used in association with subgingival re‐instrumentation in the treatment of residual periodontal pockets.^104^ In this randomized, split‐mouth, single‐blind, clinical trial, 50 subjects were treated, and the reported results indicated that after 48 weeks, the test group showed better results in terms of PD reduction (2.08 ± 1.24 vs. 1.94 ± 1.19, *p* = 0.533) and number of sites with PD ≤4 mm (38/50 vs. 35/50, *p* = 0.499), although these outcomes were not statistically significant. Similarly, CAL gain was comparable between groups (test: 0.50 ± 1.85 vs. control: 0.36 ± 1.80, *p* = 0.700). Regarding Step 3 of periodontal therapy, two studies also investigated the effect of HA combined with a minimally invasive non‐surgical technique (MINST) in the treatment of residual pockets associated with intrabony defects. The results demonstrated, respectively, a temporary clinical improvement at 3 months and an advantage in terms of reduced gingival recession.^105^, ^106^

More recently, a novel combined approach consisting of enhanced biofilm removal based on a sodium hypochlorite/amino acid solution followed by the application of a HA gel was proposed to further improve clinical outcomes.^88^, ^89^ In detail, this new antiseptic cleaning solution is based on chloramines formed after the chlorine transfer of sodium hypochlorite to the amine functions of the added amino acids.^107^, ^108^ Chloramines are able to oxidize the necrotic tissue during treatment, favoring the disruption of the biofilm and the removal of the granulation tissue. In addition, it has a softening effect on dental calculus, which makes the cleaning process easier to perform. After the completion of the mechanical debridement, a HA gel is applied into the pockets in order to improve the wound healing of the treated sites. A recent randomized controlled trial compared the clinical outcomes obtained with either mechanical subgingival debridement in conjunction with a sodium hypochlorite and amino acids containing gel (Perisolv®, Regedent AG, Zürich, Switzerland) followed by subsequent application of a cross‐linked HA gel (Hyadent® BG, Regedent AG, Zürich, Switzerland), or with mechanical debridement alone.^90^ Results showed that in terms of PD reduction and CAL gain, both groups showed statistically significant improvements at 3 and 6 months compared to baseline (*p* < 0.001) for moderate (4–6 mm) and deep pockets (≥7 mm); however, statistically significant improvements were observed in favor of the test group at both points in time (*p* < 0.001). The analysis of frequency distributions of shallow, medium, and deep pockets revealed that at baseline, subjects in the control group had 1518 (41.2%) sites with moderate pockets (4–6 mm) and the test group 1803 (48.6%) sites, respectively. At 6 months this number reduced to 803 (22.6%) in the control group and 234 (7.7%) sites in the test group, with a statistically significant difference between the groups (*p* < 0.001). Similarly, the number of deep pockets (≥7 mm) changed from 277 (7.6%) to 35 (1.0%) in control and from 298 (8.7%) to 4 (0.1%) in test at 6 months, with a statistically significant difference between the groups (*p* = 0.003). The clinical results were strengthened by the microbiological outcomes that revealed statistically significant differences in the reduction in detection frequency scores observed for all investigated bacterial species: *Aggregatibacter actinomycetemcomitans* (*p* = 0.028), *Porphyromonas gingivalis* (*p* = 0.028), *Tannerella forsythia* (*p* = 0.004), *Treponema denticola* (*p* < 0.001), and *Prevotella intermedia* (*p* = 0.003) after 6 months, favoring the test group.^109^

Finally, the effects of HA gel as an adjunct to re‐instrumentation of residual pockets in patients undergoing regular supportive periodontal care (SPC) have been investigated.^110^ Fifty‐six patients with Stages III and IV, grade B and C periodontitis, each having four interproximal residual pockets, were randomly assigned to either the test group (HA gel) or the control group (saline). After subgingival instrumentation, the assigned substance was applied subgingivally, then daily supragingivally for 3 months, and again if needed after subgingival re‐instrumentation at 3 months.

Pocket closure, defined as a probing pocket depth (PPD) of ≤4 mm with no BoP at PPD = 4 mm, was achieved in 56.8% of the experimental sites in the test group and 46.6% in the control group (*p* > 0.05). However, the median PPD and PPD distribution (5 mm) differed significantly between groups, favoring the test group at 12 months. Additionally, significantly fewer sites in the HA group required re‐instrumentation at 3 months.

**FIGURE 10:**
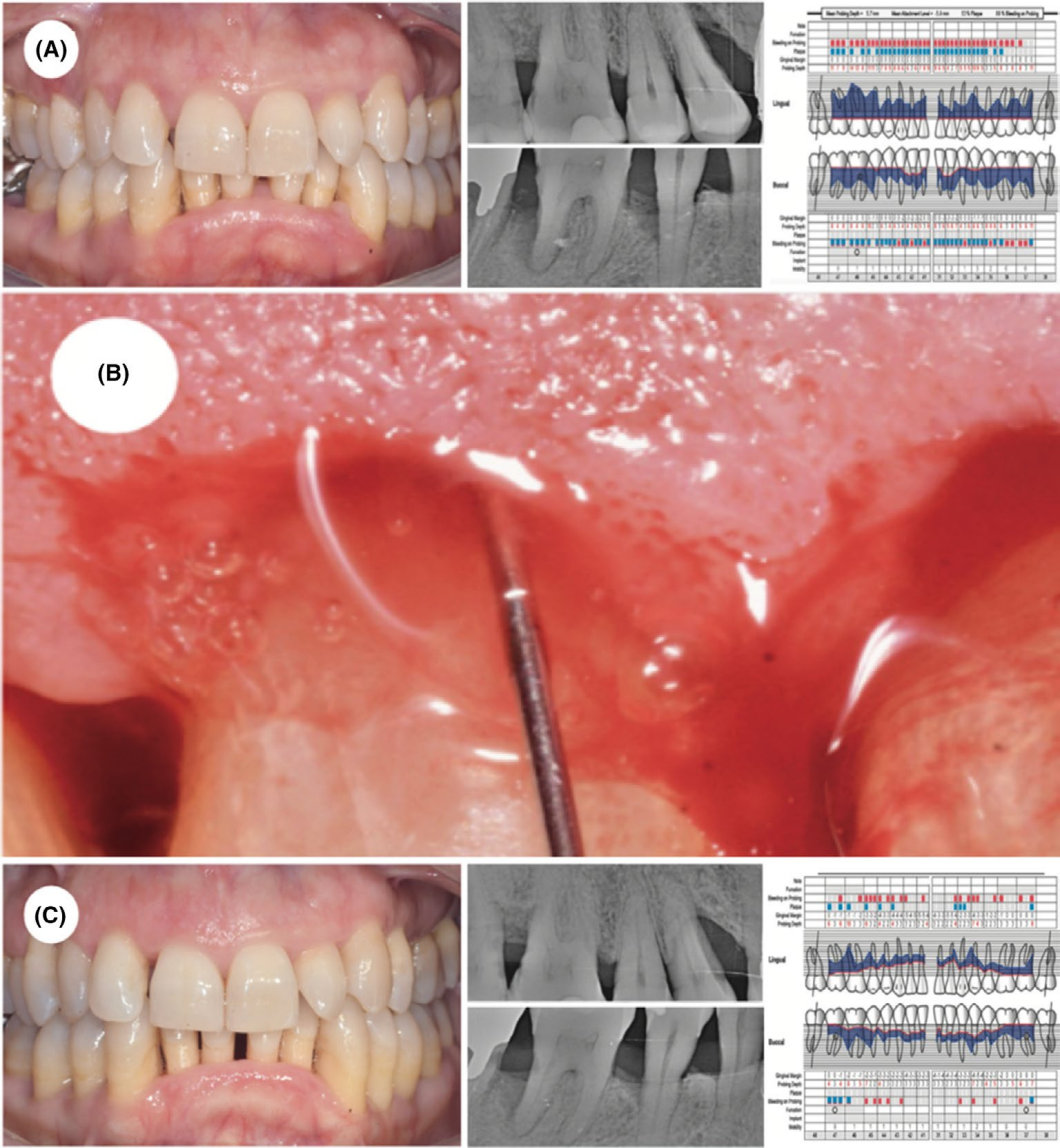
Clinical illustration of a patient 49 years old, non‐smoker, negative medical history who was affected by periodontitis (Stage 3, grade C) and referred for periodontal treatment. (A) Presence of both horizontal and vertical bone resorption (>2 mm) in correspondence with Teeth 1.4, 2.4, 2.6, 2.7, 3.4, 3.5, 3.6, 4.6. Tissue phenotype was thick (>1 mm). Periodontal data recorded at baseline showed an average probing depth of 5.7 mm, average attachment level of −5.9 mm, plaque index of 53%, and % of bleeding on probing of 88%. (B) A gel containing Polynucleotides and hyaluronic acid was used after a thorough subgingival instrumentation. (C) The periodontal charting 6 months after therapy showed an average pocket depth of 3.4 mm, average attachment level of −4.8 mm, plaque index of 11%, and % bleeding on probing of 34% (average reduction in pocket depth: 2.3 mm, average gain in attachment level: 1.1 mm, average reduction in plaque: 42%, average reduction in bleeding: 54%).

Figure 10 illustrates the clinical use of HA in non‐surgical periodontal therapy.

In conclusion, different formulations and combinations of HA have also been tested in several studies, and its biochemical and clinical characteristics have demonstrated numerous potential benefits to be used as a valid adjuvant in non‐surgical periodontal therapy.

### 7.3 Clinical outcomes in the surgical treatment of intrabony defects

Over the past 20 years, increasing research has underscored the potential of using HA in conjunction with regenerative periodontal surgery. The first use of HA in periodontal surgery of periodontal pockets associated with intrabony defects was reported by Engström et al. in 2001.^111^ In this controlled, split‐mouth clinical study, they treated six individuals exhibiting two teeth with comparable bony defects associated with a probing depth ≥ 6 mm. For each patient, one tooth was assigned to receive guided tissue regeneration (GTR) using a bioresorbable membrane plus locally delivered HMW HA (test site) and the other tooth GTR alone (control site). At baseline, 2 weeks, and 1, 3, 6, and 12 months after treatment, alveolar bone height and bone healing patterns were analyzed using digital intraoral radiographs. PPD, BoP, and the presence of plaque were recorded, a microbiological examination was performed, and gingival crevicular fluid was sampled for analysis of immunoglobulin G, C3, and prostaglandin E2 levels. Except for a less than 1 mm increase in bone height in the test sites after 12 months, all other clinical, immunological, and microbiological variables were comparable. A few years later, Ballini et al. (2009) and Vanden Bogaerde (2009) conducted two prospective case series on the effects of periodontal surgery using autologous bone mixed with a low molecular weight esterified HA preparation and esterified HA alone, respectively.^112^, ^113^ In the first study, the authors reported the results of 9 patients with intrabony defects that showed a mean increase in clinical attachment level of 2.6 mm at 24 months, confirmed by radiographic evaluation. In the second study, the results were reported 12 months after the treatment of 18 infrabony furcations and one mandibular molar furcation. The mean values of the reduction in PPD were 5.8 mm, with an increase in gingival recession of 2.0 mm and the clinical attachment gain of 3.8 mm.

**FIGURE 11:**
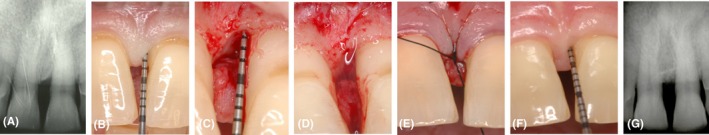
Clinical illustration of a residual periodontal pocket associated with an intrabony defect treated by means of a single flap approach in conjunction with HA alone. (A) baseline radiographic view; (B) baseline clinical view; (C) intraoperative view of the intrabony defect; (D) hyaluronic acid application; (E) suture; (F) 24‐month clinical follow‐up; (G) 24‐month radiographic follow‐up.

Subsequently, the effectiveness of HA in the surgical treatment of intrabony defects compared to open flap debridement (OFD)/modified Widman flap (MWF) alone was investigated by a series of RCTs. The first in this series was a randomized split‐mouth study published by Fawzy El‐Sayed et al.,^114^ which included 14 patients with chronic periodontitis, each of whom had four interproximal intrabony defects (≥3 mm) with PPD values > 5 mm. All defects were treated by MWF surgery and two of them received an application of 0.8% HMW HA gel (test sites) while the others received placebo gel (control). At baseline and at 3 and 6 months, CAL, PPD, gingival recession (GR), plaque index (PI) and BOP were measured. Statistically significant differences were observed at both test and control sites compared to baseline. This study reported for the first time, unlike Engström et al.^111^ where no differences were observed for any clinical parameter, test sites showed a higher CAL increase and GR reduction than control sites. This finding suggested that sites treated with HA achieved the same PD reduction but presented a limited increase in GR. Subsequently, Briguglio et al.^115^ designed an RCT to examine the clinical effects of OFD with or without the use of HA to treat intrabony periodontal defects over a 24‐month period. Forty subjects with a two‐walled intrabony defect (PPD ≥7 mm; CAL ≥7 mm) were selected and divided into two groups: 20 patients were treated with OFD and LMW HA (test group) and 20 patients with OFD only (control group). At 12 and 24 months, PI, BOP, PPD, and CAL were measured. Contrary to the previous study, at the latest follow‐up, the defects showed a statistically significant better clinical response in terms not only of CAL (1.9 ± 1.8 mm vs. 1.1 ± 0.7 mm) but also of PPD (1.6 ± 1.2 mm vs. 0.8 ± 0.5 mm), showing no differences in the position of the gingival margin between the groups. More recently, Mamajiwala et al.^116^ performed a randomized, controlled, split‐mouth, clinical trial including 20 chronic periodontitis patients having at least two contralateral intrabony defects. Defects (20 in each group) were randomly divided into test (0.8% HA gel + OFD) and control (OFD + placebo) groups. Clinical parameters assessed at baseline, 6 months, and 12 months were PI, gingival index (GI), PPD, CAL, and GR. Unlike previous authors who evaluated bone fill using periapical radiography but did not report any quantitative evaluation, in the present investigation cone beam computed tomography (CBCT) was used to evaluate bone defect (DF) filling, changes of the alveolar ridge (ACC) and resolution of the defect at baseline and 12 months. Compared to previous studies, an even more pronounced beneficial clinical effect of HA was demonstrated compared to OFD alone (CAL gain: 5.1 ± 1.2 vs. 4.05 ± 1.19 mm; PPD reduction 5.3 ± 1.2 vs. 4.35 ± 0.81 mm; GR increase: 0.7 ± 0.73 vs. 1.2 ± 0.76 mm). Furthermore, bone defect filling (DF) was 5.67 ± 2.01 versus 4.49 ± 1.78 mm. Regenerative surgical therapy of a single intrabony defect using HA alone is presented in Figure 11.

The use of HA alone in regenerative periodontal surgery was also compared with one of the gold standard therapeutic options for the treatment of residual pockets associated with intrabony defects, namely enamel matrix derivatives.^117^ In detail, 32 intrabony defects in 32 patients were randomly assigned to receive a single‐flap approach in combination with cross‐linked HMW HA (test group) or an enamel matrix derivative (control group). At 24 months, the results of this randomized, controlled clinical trial showed statistically comparable results in terms of CAL gain (2.19 ± 1.11 mm in the test and 2.94 ± 1.12 mm in the control sites). Interestingly, the control sites showed statistically significantly higher PPD reduction. However, lower GR values were observed in HA‐treated sites. These results were corroborated by a clinical and radiographic 6‐month prospective trial.^118^

**FIGURE 12:**
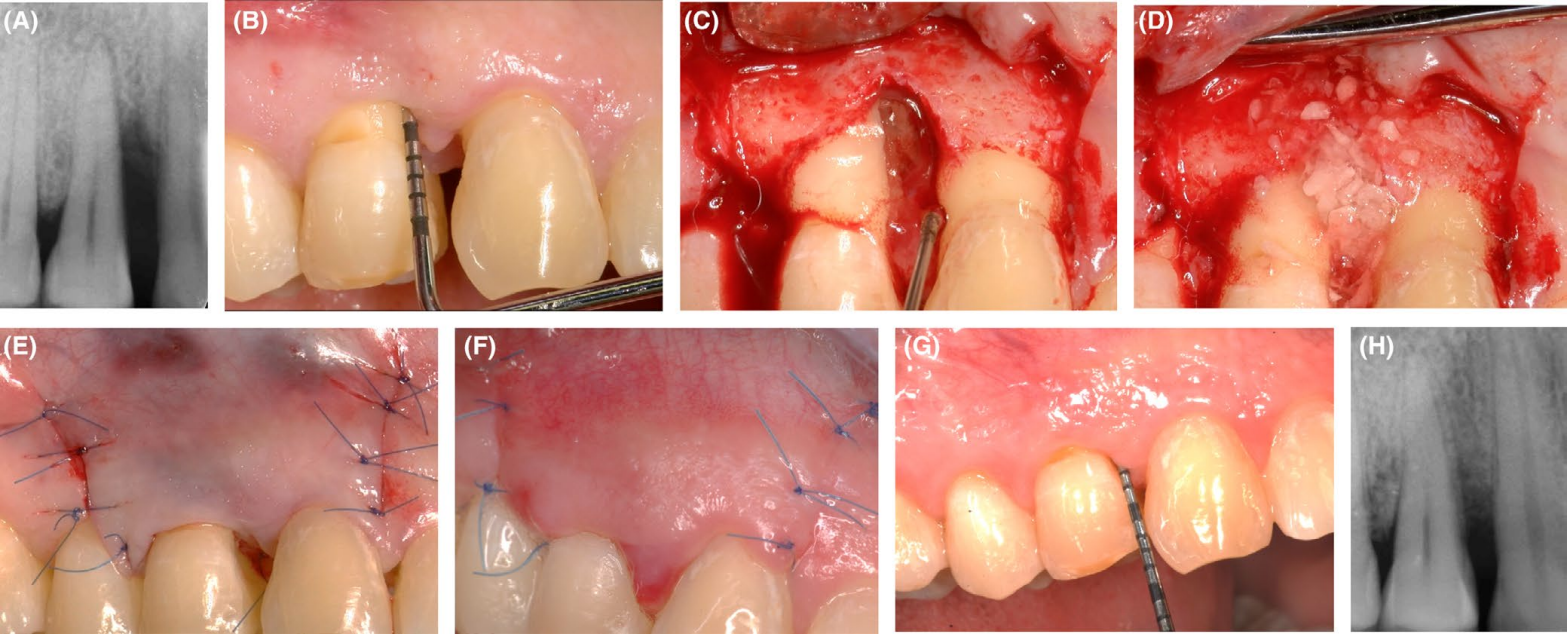
Clinical illustration of a residual periodontal pocket associated with an intrabony defect treated by means of a single flap approach in conjunction with HA and a bone substitute. (A) baseline radiographic view; (B) baseline clinical view; (C) intraoperative view of the intrabony defect and HA application; (D) bone substitute application; (E) suture; (F) early wound healing at 2 weeks; (G) 24‐month clinical follow‐up; (H) 24‐month radiographic follow‐up.

After Engström et al. (2001), Ballini et al. (2009) and Vanden Bogaerde (2009),^111^, ^112^, ^113^ other research groups have focused their attention on the role of HA when used in combination with other regenerative materials such as barrier membranes or bone substitutes. Sehdev et al.^119^ performed a randomized controlled clinical trial to test the effect of HA in combination with a bioresorbable membrane (test group) compared to a bioresorbable membrane alone (control group) in the treatment of 24 intrabony defects in 20 systemically healthy patients. Pocket probing depth (PPD), relative attachment level, relative gingival margin level, and radiographic parameters were measured at baseline and 6 months. The test group showed a CAL gain of 2.20 mm also associated with a statistically significant higher PPD reduction (4.52 vs. 2.97 mm). The reduction in defect depth in the test group was 3.92 mm with a defect filling of 94%, and in the control group, it was 2.08 mm with a defect filling of 55.55%. In 2021, Božić et al.^120^ reported the results of the treatment of 23 patients with 27 intrabony defects by means of papilla preservation techniques in combination with cross‐linked HMW HA and deproteinized bone mineral. In this case series, mean CAL gain and mean PPD reduction at 6 months were 3.65 and 4.54 mm, respectively, and they were associated with a mean GR increase of 0.89. In the same year, Bhowmik and Rao^121^ published the results of a randomized, controlled split‐mouth trial comparing the efficacy of a hyaluronan‐nanohydroxyapatite composite graft (test sites) versus a nano hydroxyapatite graft (control sites) in the management of 10 patients presenting with 38 intrabony defects. At 12 months, test sites showed greater PPD reduction (5.06 mm vs. 3.21 mm), CAL gain (4.00 mm vs. 3.21 mm) together with a better improvement in radiographic parameters. Regenerative surgical therapy of an intrabony defect using HA in combination with a bone substitute is presented in Figure 12.

Finally, HA has been used as a biologic agent in regenerative therapy as part of gels containing other molecules. Specifically, de Santana et al.^122^ investigated in a controlled split‐mouth study whether a biologic hydrogel of a recombinant human fibroblast growth factor type 2 (rhFGH) in an HA carrier applied in the regenerative treatment of 30 patients who had two intraosseous defects each would improve the clinical results compared to OFD with papilla preservation only. After 1 year, test sites showed significantly greater PD reduction (5.5 mm vs. 2.9 mm), increased CAL (4.8 mm vs. 2.2 mm), and a lower percentage of residual PPD > 5 mm (0% vs. 100%). More recently, a gel containing a mixture of polynucleotides and HA (PN‐HA) was used alone or in combination with deproteinized bovine bone mineral with papillary preserving flaps in the treatment of 43 patients exhibiting 55 intrabony defects.^123^ At 12 months, the reduction in PPD was 4.4 ± 1.8 mm with an increase in GR of 1.0 ± 0.8 mm. Furthermore, radiographic bone fill was 3.5 ± 1.9 mm.

These findings highlight the potential for HA, used either alone or in combination with various grafting materials, to enhance the clinical outcomes in surgical regenerative periodontal therapy, offering promising avenues for periodontal intrabony defects management.

Table 1 summarizes the studies on the use of HA in regenerative therapy.

**TABLE 1:** Summary of the studies on the use of HA in regenerative therapy.

	Study design	Patients (test/control IBDs)	Follow‐up (months)	Intervention	Baseline PD (mm)	Follow‐up PD (mm)	Baseline CAL (mm)	Follow‐up CAL (mm)	Radiographic bone change
Engström et al. (2001)	RCT Split‐mouth	6 (6/6)	12	GTR + HMW HA gel	7.8 ± 1.1	3.8 ± 0.7	NR	NR	Radiographic bone height increase of 2.2%
				GTR	7.3 ± 0.9	4.3 ± 1.4	NR	NR	Radiographic bone height decrease of 1.8%
Ballini et al. (2009)	Prospective case series	9 (9)	24	OFD + autologous bone + LMW HA fibers ester	6.75 ± 1.12	3.3 ± 0.77	5.0 ± 0.84	1.59 ± 1.02	No values of observed radiographic bone fill are reported
Vanden Bogaerde (2009)	Prospective case series	19	12	OFD + LMW HA fibers ester	9.84 ± 2.32	4.0 ± 1.41	10.3 ± 2.29	6.53 ± 2.06	NR
Fawzy El‐Sayed et al. (2011)	RCT Split‐mouth	14 (14/14)	6	MWF + HMW HA gel	5.0 (5.00/6.00)	3.0 (2.00/4.00)	5.0 (2.00/7.00)	2.0 (1.00/3.50)	NR
				MWF	5.0 (5.00/6.00)	3.0 (2.00/4.00)	5.0 (2.00/8.00)	3.0 (2.00/5.00)	NR
Briguglio et al. (2013)	RCT Double‐arm	40 (20/20)	24	OFD + LMW HA fibers ester	8.6 ± 1.5	7.0 ± 1.2	7.20 ± 1.50	5.30 ± 1.80	No values of observed radiographic bone gain are reported
				OFD	8.0 ± 0.7	7.2 ± 0.5	8.30 ± 1.20	7.20 ± 0.70	
de Santana et al. (2015)	RCT Split‐mouth	30 (30/30)	12	OFD with PPF + HA/ rhFGF‐2	9.7 ± 1.9	4.2 ± 0.8	10.4 ± 1.6	5.7 ± 1.4	NR
				OFD with PPF	9.5 ± 1.5	6.6 ± 1.3	10.3 ± 1.3	8.0 ± 1.9	NR
Sedhev et al. (2016)	RCT	20 (12/12)	6	GTR + LMW HA fibers ester	6.40 ± 0.80	1.88 ± 0.3	13.81 ± 0.80	9.33 ± 0.35	Radiographic bone fill increase of 94.00 ± 8.74%
				GTR	NR	**−2.97 ± 0.85** ^a^	13.68 ± 0.7	11.40 ± 0.3	Radiographic bone fill increase of 55.55 ± 13.18%
Mamajiwala et al. (2021)	RCT Split‐mouth	20 (20/20)	12	OFD + HMW HA gel	8.50 ± 0.94	3.1 ± 0.58	9.15 ± 0.48	4.0 ± 0.56	Radiographic defect fill of 5.67 ± 2.01
				OFD	8.45 ± 0.51	4.3 ± 0.47	9.30 ± 0.73	5.4 ± 0.82	Radiographic defect fill of 4.49 ± 1.78
Pilloni et al. (2021)	RCT Double‐arm	32 (16/16)	24	SFA + HMW HA gel	7.31 ± 0.97	4.00 ± 1.09	7.37 ± 0.88	5.19 ± 1.42	NR
				SFA + EMD	7.25 ± 0.93	2.75 ± 0.57	7.37 ± 0.96	4.44 ± 1.03	NR
Bozic et al. (2021)	Prospective case series	23 (27)	6	DPBM + HMW HA gel	7.89 ± 1.60	3.35 ± 0.72	8.72 ± 1.82	5.07 ± 1.82	NR
Bhowmik et al. (2021)	RCT Split‐mouth	8 (16/16)	12	NHA + HMW HA gel	7.06 ± 1.34	2.00 ± 0.76	5.33 ± 1.60	1.33 ± 1.18	Radiographic defect depth reduction of 48.22 ± 31.56%
				NHA	6.71 ± 1.13	3.50 ± 1.78	5.57 ± 1.45	2.71 ± 1.32	Radiographic defect depth reduction of 20.14 ± 25.34%
Cairo et al. (2024)	Prospective case series	43 (55)	12	PPT + PN‐HA alone or in combination with DBBM	7.7 ± 1.9	3.2 ± 0.7	9.6 ± 2.4	6.1 ± 1.7	Radiographic bone fill of 3.5 ± 1.9 mm
Vela et al. (2024)	RCT Double‐arm	54 (27/27)	6	OFD + HMW HA	5.9 ± 2.0	3.8 ± 1.7	9.1 ± 2.3	5.9 ± 1.8	Comparable radiographical improvements
				OFD + EMD	5.7 ± 1.9	3.8 ± 1.1	8.9 ± 2.2	5.8 ± 1.7	

Abbreviations: CAL, clinical attachment loss; DBBM, deproteinized bovine bone mineral; DPBM, deproteinized porcine bone mineral; GTR, guided tissue regeneration; HA, hyaluronic acid; HMW, high molecular weight; IBDs, intrabony defects; LMW, low molecular weight; MWF, modified Widman flap; NHA, nano hydroxyapatite; NR, not reported; OFD, open flap debridment; PD, probing depht; PN‐HA, polynucleotides and hyaluronic acid; PPF, papilla preservation flap; PPT, papilla preservation technique; randomized control trial; rhFGF‐2, human recombinant basic fibroblast growth factor 2RCT; SFA, single flap approach.

^a^ Only PD reduction value available.

### 7.4 Clinical outcomes in mucogingival therapy

HA could be regarded as a valuable adjunct in various surgical approaches aimed at treating mucogingival defects. Indeed, it can be employed in the management of single or multiple gingival recessions, utilizing techniques such as the coronally advanced flap (CAF), with or without a connective tissue graft. Moreover, HA could also be considered when performing modified coronally advanced tunnel or laterally closed tunnel techniques, both commonly using subepithelial connective tissue grafts.

**FIGURE 13:**
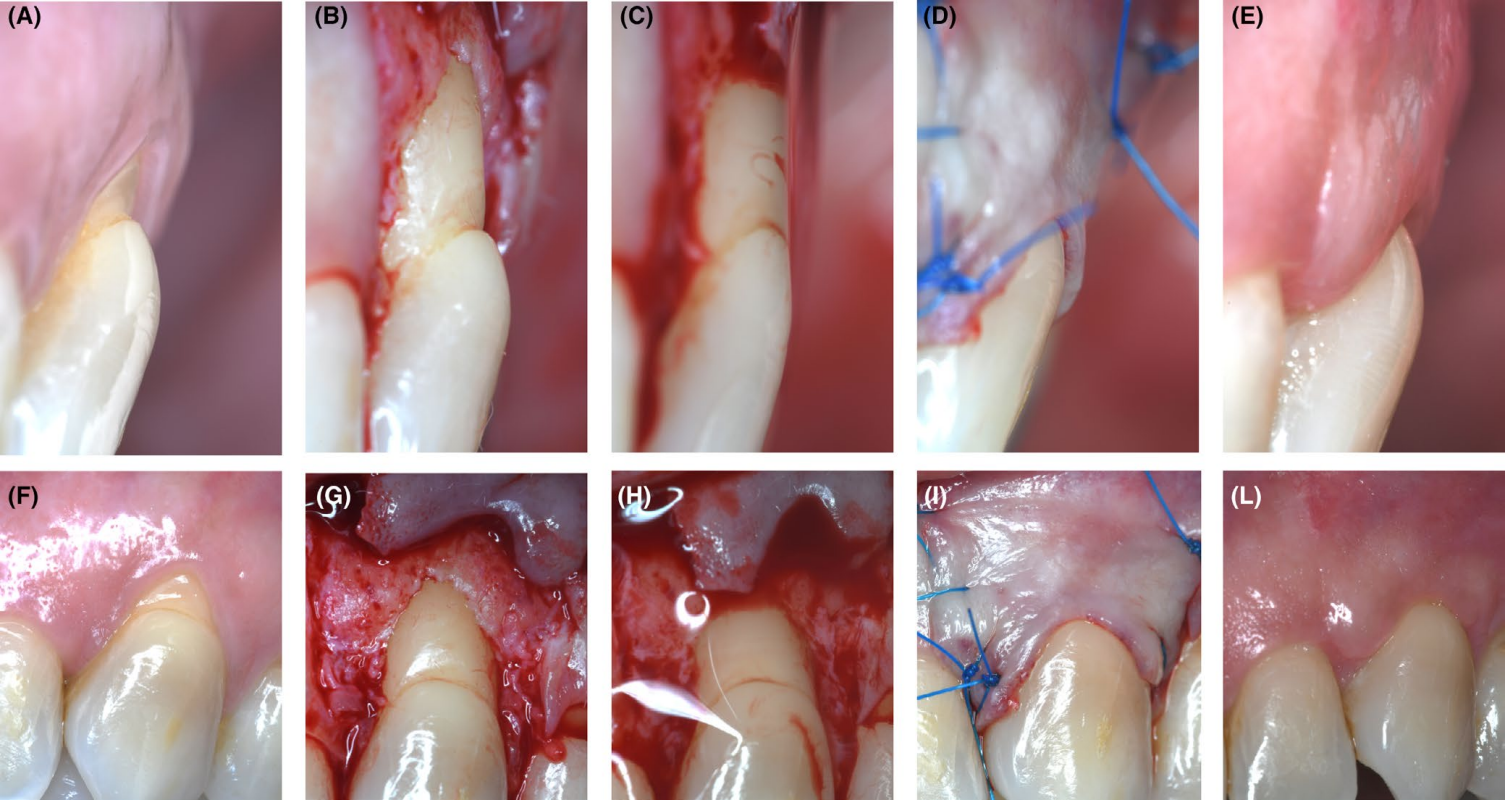
Clinical illustration of a single type 1 (RT1) gingival recession with >2 mm of keratinized tissue treated by means of coronally advanced flap in combination with HA from a lateral (A–E) and frontal (F–L) view. (A and F) baseline; (B and G) split‐full‐split‐thickness flap elevation; (C and H) application of HA gel; (D and I) suture; (E and L) clinical follow‐up at 6 months.

**FIGURE 14:**
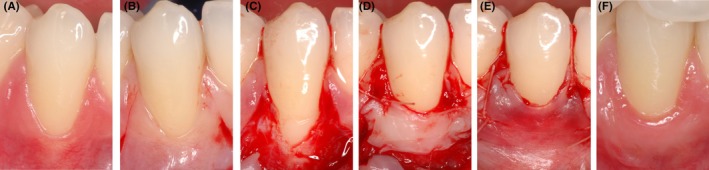
Clinical illustration of a single type 1 (RT1) gingival recession with <2 mm of keratinized tissue treated by coronally advanced flap and connective tissue graft in combination with HA. (A) baseline; (B) flap design; (C) split‐full‐split‐thickness flap elevation and HA gel application; (D) suturing of connective tissue graft; (E) flap suture; (F) clinical follow‐up at 24 months.

**FIGURE 15:**
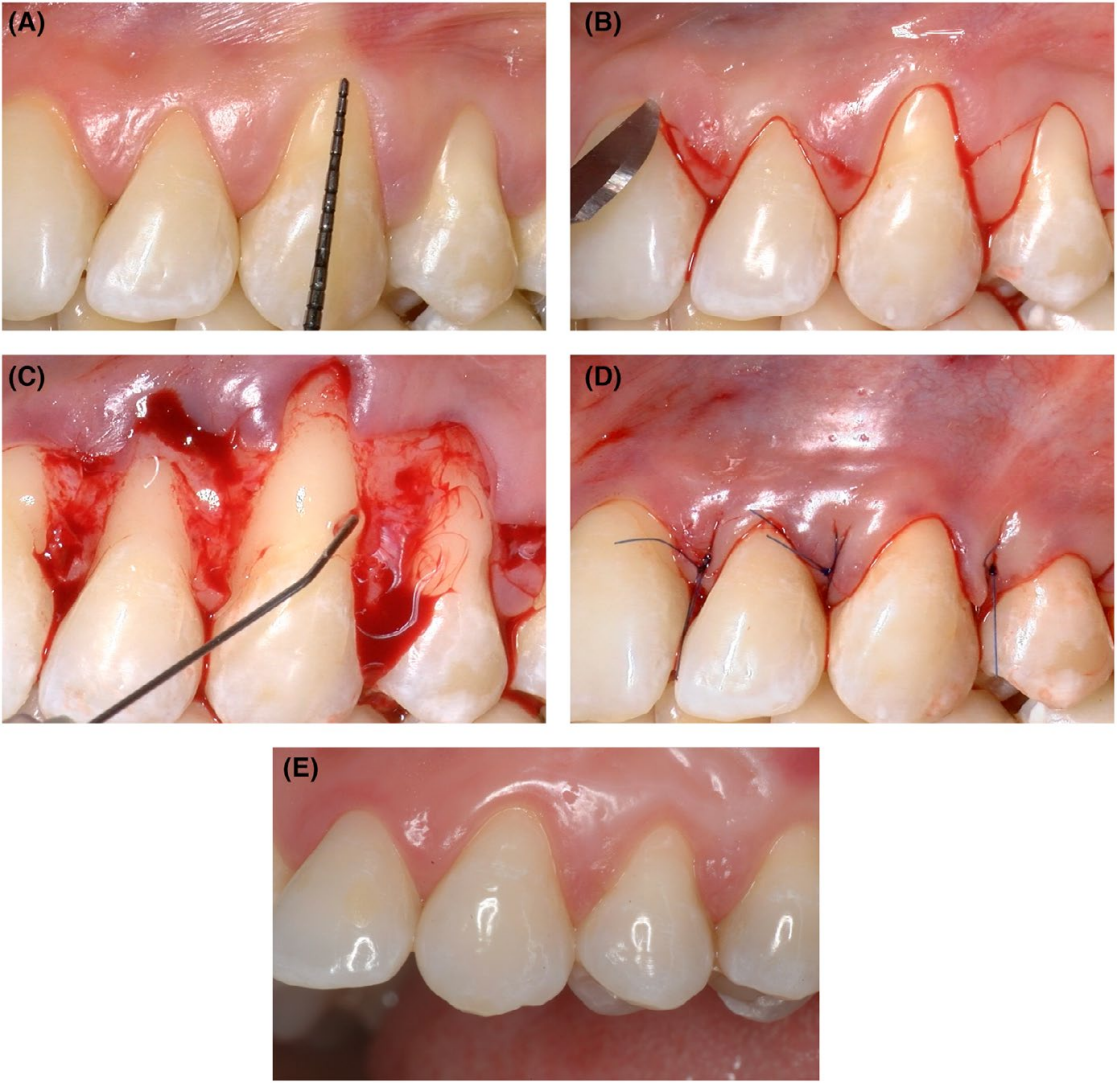
Clinical illustration of a multiple type 1 (RT1) gingival recession treated by coronally advanced flap in combination with HA. (A) baseline; (B) flap design; (C) split‐full‐split‐thickness flap elevation and HA gel application; (D) flap suture; (E) clinical follow‐up at 24 months.

**FIGURE 16:**
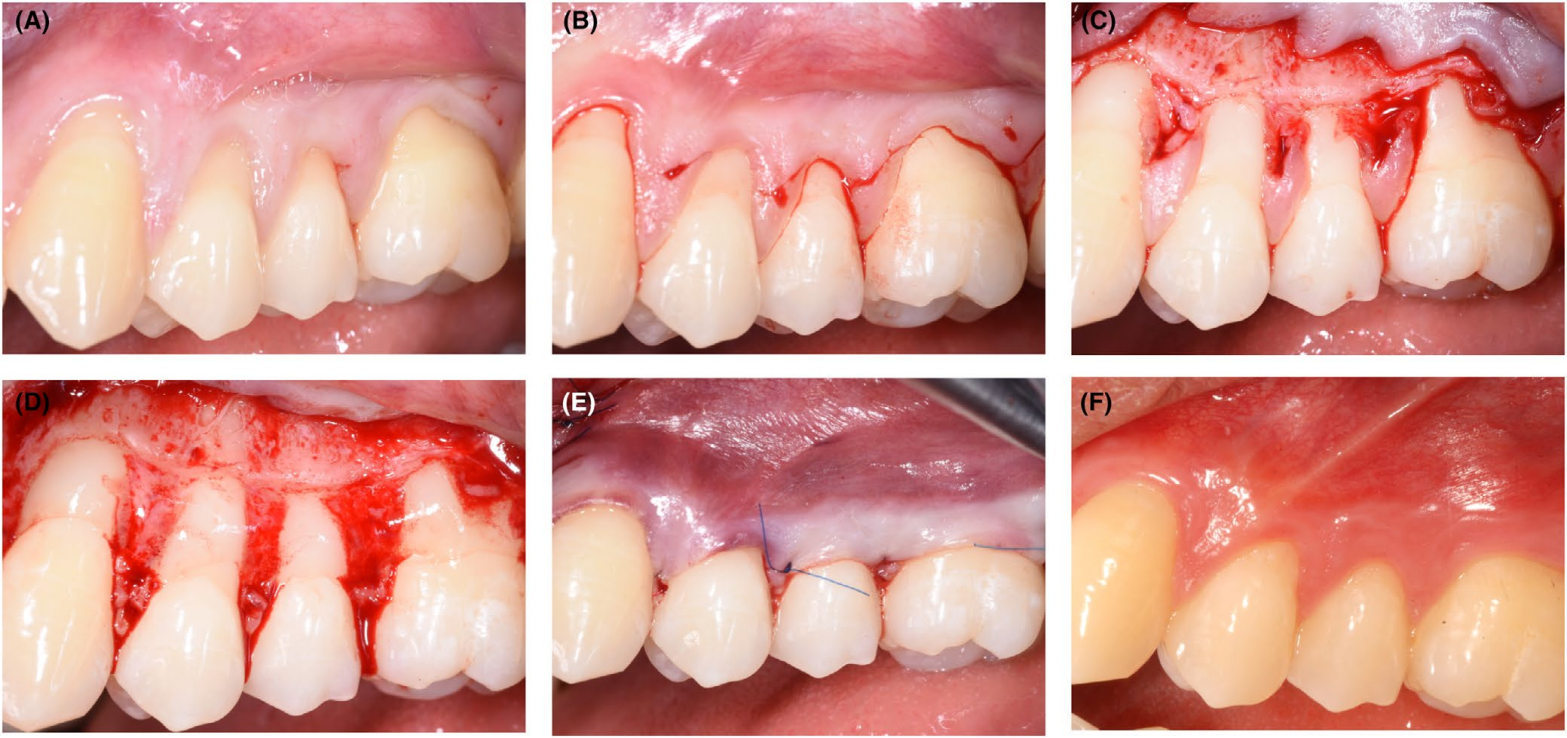
Clinical illustration of a multiple type 1 (RT1) gingival recessions treated by coronally advanced flap in combination with HA. (A) baseline; (B) flap design; (C) split‐full‐split‐thickness flap elevation; (D) HA gel application; (E) flap suture; (F) clinical follow‐up at 24 months.

**FIGURE 17:**
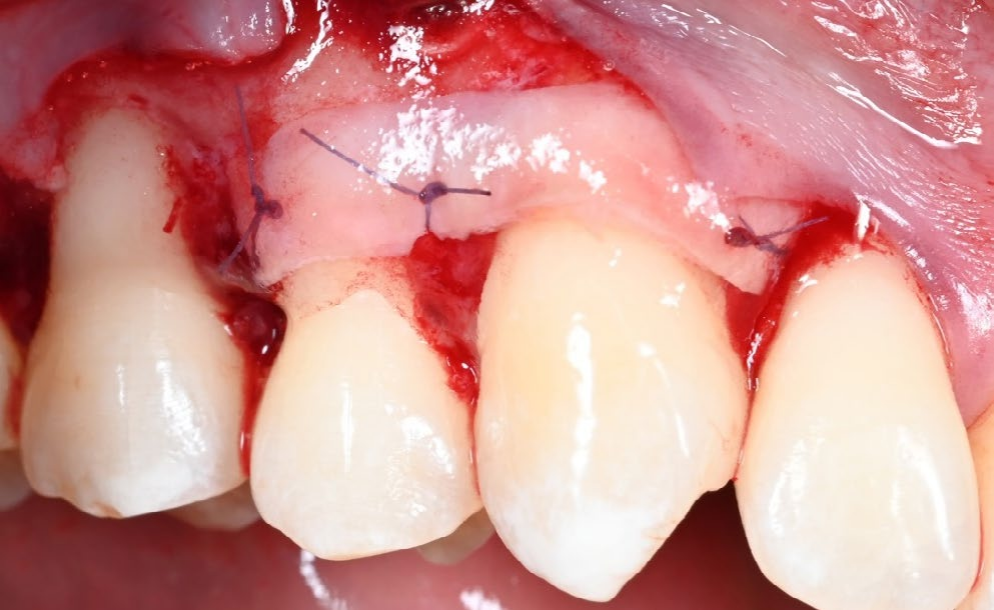
Intra‐operative application of HA as an adjunct to coronally advanced flap and connective tissue graft for multiple gingival recession coverage.

The use of HA in the treatment of recession type 1 by means of CAF was firstly described by Kumar et al.^124^ In this split‐mouth controlled clinical trial, 10 patients showing 20 Miller class I (recession type 1) recessions were treated using a CAF with or without the application of a HMW HA gel on root surfaces before suture application. At 24 weeks, the authors did not report statistically significant differences in the change of recession depth (RD) reduction (2.1 mm vs. 1.9 mm) and mean root coverage (MRC) (68.33 vs. 61.67%) between test and control sites. In 2015, Rajan et al.^125^ randomly assigned 20 patients with a minimum of two Miller class I and II (recession type 1) gingival recessions to receive a CAF combined with HA gel (test sites) or sub epithelial connective tissue graft (sCTG) (control sites). At 9 months, sites treated with CAF and HA showed no statistical difference in RD reduction (2.60 mm vs. 2.30 mm), MRC (77.84% vs. 82.15%) and keratinized tissue (KT) width (3.20 mm vs. 3.30 mm) compared to sites treated with CAF and sCTG. Unlike Kumar et al. (2014), Pilloni et al. (2019) reported a benefit deriving from the application of HMW HA combined with CAF in the treatment of a single Miller class I gingival recession (Type 1 recession) compared to CAF alone.^126^ In fact, the results of this randomized controlled trial with parallel arms including 15 patients per group showed at 18 months a statistically significant greater RD reduction (2.7 mm vs. 1.9 mm) and MRC (93.8% vs. 73.1%) in the group treated with CAF + HA compared to the control group. Furthermore, it was interesting to note that postoperative discomfort and swelling, as measured by a visual analog scale, were statistically less in the test group. However, no differences in KT change were observed between groups. The adjunctive use of HA to CAF in the treatment of single and multiple type 1 gingival recessions with and without connective tissue grafts is presented in the clinical cases shown in Figures 13, 14, 15, 16, 17.

**FIGURE 18:**
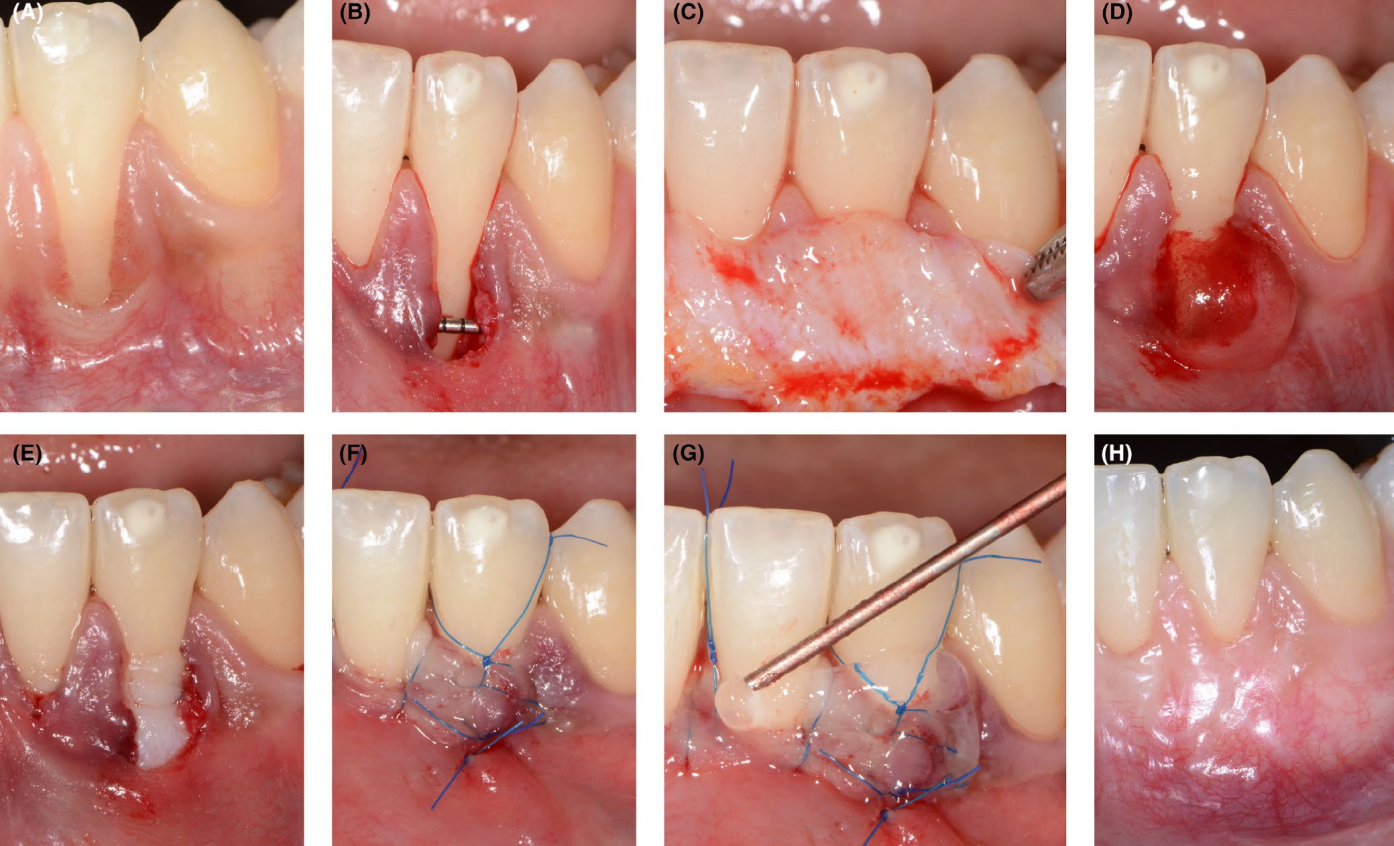
Clinical case depicting the treatment of a 35‐year‐old, non‐smoker, systemically healthy, female patient exhibiting a deep RT1 recession located in the mandibular frontal area using the Laterally Closed Tunnel (LCT)^127^ in conjunction with a cross‐linked HA and a palatal subepithelial connective tissue graft (SCTG). (A) Preoperative situation, (B) Prepared tunnel, (C) Harvested CTG, (D) Application of HA, (E) Sutured SCTG, (F) Closed tunnel, (G) Application of HA on the closed tunnel, (H) Clinical outcome at 1 year indicating complete root coverage.

More recently, the results of two prospective case series on the treatment of gingival recession using coronally advanced modified tunnel or laterally closed tunnel and sCTG techniques in combination with HMW HA were presented. In the first study, 12 patients with a single type 1 recession were included.^127^ At 6 months, a reduction in RD of 4.1 mm, an increase of KT of 3.3 mm, a MRC of 96.09%, and a complete root coverage (CRC) of 50% were reported. In the second study, 15 patients with multiple gingival recession types 1 and 2 were included.^128^ At the follow‐up visit (from 6 to 33 months), the reduction of RD, the increase of KT (MRC and CRC) were 2.5 mm, 1.2 mm, 85.1%, and 20%, respectively. In this study, the aesthetic outcome, reported using the aesthetic root coverage score (RES), was 7.9 ± 1.9, indicating good patient satisfaction. Figure 18 illustrates the clinical use of HA in conjunction with the laterally closed tunnel and sCTG.

Despite the limited number and heterogeneity of studies available in the literature, HA appears to improve the results of mucogingival therapy. However, more robust randomized controlled trials are needed to clarify the potential advantage of using HA in association with different surgical approaches for the treatment of gingival recessions.

Table 2 summarizes the studies on the use of HA in mucogingival therapy.

**TABLE 2:** Summary of the studies on the use of HA in mucogingival therapy.

	Study design	Sample	Recession type	Follow‐up (months)	Intervention	Baseline RD (mm)	Follow‐up RD (mm)	MRC	CRC
Kumar et al. (2014)	RCT Split‐mouth	10	Single RT1	6	CAF + HA	3.20	1.1	68.3%	40.0%
					CAF	2.90	1.0	61.6%	20.0%
Rajan et al. (2015)	RCT Split‐mouth	20	Single RT1	9	CAF + HA	3.65	1.05	77.84%	NR
					CAF + sCTG	3.45	1.15	82.15%	NR
Pilloni et al. (2019)	RCT Double‐arm	30 (15/15)	Single RT1	18	CAF + HA	3.0	0.0	93.8%	80.0%
					CAF	3.0	1.0	73.1%	30.0%
Guldener et al. (2020)	Case series	12	Single RT1	6–30	MCAT + sCTG + HA or LCT + sCTG + HA	4.6	0.5	96.09%	50.0%
Lanzrein et al. (2020)	Case series	15	Multiple RT1 and RT2	6–33	MCAT + sCTG + HA or LCT + sCTG + HA	3.3	0.8	85.1%	20.0%

Abbreviations: CAF, coronally advanced flap; CRC, complete root coverage; HA, hyauuronic acid; LCT, laterally closed tunnel; MCAT, modified coronally advanced tunnel; MRC, mean root coverage; NR, not reported; RCT, randomized controlled trial; RD, recession depth; RT, recession type; sCTG, sub‐epithelial connective tissue graft.

## 8 CONCLUSIONS AND OUTLOOK IN THE FUTURE

The available data from preclinical and clinical studies provide robust evidence on the effects of HA to enhance periodontal wound healing and regeneration, and on the improved clinical outcomes when HA is used in conjunction with nonsurgical periodontal therapy and regenerative surgery in intrabony and recession defects. More consistent and compelling results have been observed in regenerative surgical procedures, where the reviewed studies demonstrate HA's potential to enhance CAL gain, reduce PPD, and minimize gingival recession compared to conventional treatments. These findings support the central hypothesis that HA is a valuable adjunct in periodontal regenerative/reconstructive surgeries, contributing to better wound healing outcomes. However, due to some heterogeneity and variable statistical significance among studies, further research is needed to establish standardized protocols for HA application. Furthermore, additional studies are warranted to explore the biological and clinical relevance of various HA formulations used alone or in combination with bone grafts/bone substitutes, soft tissue grafts, and growth factors for regenerative treatment at teeth and in the treatment of peri‐implant mucositis and peri‐implantitis.

## 9 FINAL REMARKS

Since the early 1980s, research regarding tissue healing and regeneration around teeth and implants considering different cellular, biomolecular, and microbiological aspects has been performed. When exploring the potential of hyaluronic acid, both basic and clinical studies have demonstrated that HA participates in almost all the aforementioned mechanisms and has clinical benefits. However, it seems that dentistry has come to these conclusions on HA quite late considering the many decades of solid data on the crucial roles of HA in tissue healing.

The 1981 Nobel Prize Roger Sperry once quoted, “Every single cell of your body is designed to reproduce, recreate and rejuvenate itself. That's what life does, that's how it works…if your body receives proper nutrition, hydration and movement then your cells will have the energy to vibrate with health and express themselves…”.

## FUNDING INFORMATION

The study was self‐funded by the authors and their institutions.

## CONFLICT OF INTEREST STATEMENT

The authors declare no conflicts of interest in connection with this article.

## Data Availability

Data sharing not applicable to this article as no data sets were generated or analyzed during the current study.
